# Does Shift in Vegetation Abundance After Nitrogen and Phosphorus Additions Play a Key Role in Regulating Fungal Community Structure in a Northern Peatland?

**DOI:** 10.3389/fmicb.2022.920382

**Published:** 2022-06-09

**Authors:** Chenhao Cao, Jingjing Huang, Leming Ge, Tong Li, Zhao-Jun Bu, Shengzhong Wang, Zucheng Wang, Ziping Liu, Shasha Liu, Meng Wang

**Affiliations:** ^1^Key Laboratory of Geographical Processes and Ecological Security in Changbai Mountains, Ministry of Education, School of Geographical Sciences, Northeast Normal University, Changchun, China; ^2^State Environmental Protection Key Laboratory of Wetland Ecology and Vegetation Restoration, Institute for Peat and Mire Research, Northeast Normal University, Changchun, China; ^3^Jilin Provincial Key Laboratory for Wetland Ecological Processes and Environmental Change in the Changbai Mountains, Changchun, China; ^4^Center for Ecological Forecasting and Global Change, College of Forestry, Northwest A&F University, Xianyang, China; ^5^School of Geographic Sciences, Hunan Normal University, Changsha, China

**Keywords:** mycorrhizae, bog, fen, Ericaceae, deposition, Changbai Mountains

## Abstract

Soil fungal communities are key players in biogeochemical processes of peatlands, which are important carbon stocks globally. Although it has been elucidated that fungi are susceptible to environmental changes, little is known about the intricate and interactive effect of long-term nitrogen (N) and phosphorus (P) enrichment on fungal community structure in northern peatlands. In this study, we compared a short- (2 years) with a long-term (10 years) fertilization experiment in a peatland complex in northeastern China to assess how N and/or P additions influence fungal community structure. The results showed that fungal community composition and diversity were altered by N addition, without a significant interactive effect with P addition. Not only the long-term but also the short-term nutrient addition could change the abundance of different plant functional types. However, there were no strong cascading effects on the fungal community in any of the fertilization experiments. Long-term nutrient addition showed a stronger effect on the relative abundance of different fungal functional guilds; an increase in the relative abundance of saprotrophs after fertilization did not jeopardize mycorrhizal fungi. Moreover, the decline in *Sphagnum* cover after long-term N addition did not parallel changes in the relative abundance of *Sphagnum*-associated fungi (*Clavaria sphagnicola*, *Galerina tibiicystis*, *G. sphagnicola*, and *G. paludosa*). Given that short- and long-term fertilization showed strongly contrasting effects on fungal community structure, our study highlights the necessity of assessing the long-term effects of nutrient enrichment on the association between vegetation and fungal community in peatland ecosystems. Future research priorities should emphasize the connection between the community structure of fungal functional guilds and their functionality, which is of paramount importance to better understand their influences on C storage in the face of uncertain N and P deposition regimes.

## Introduction

Northern peatlands are important global carbon (C) sinks, storing one-third of global soil organic C (500 ± 100 Gt), while occupying ∼3% of Earth’s surface (Yu et al., 2010; Loisel et al., 2014). Among the important factors that limit plant productivity and organic matter decomposition in peatlands, for example, anaerobic, frigid, and nutrient-deficient soil environments (Clymo, 1984; Rydin and Jeglum, 2013), the low nitrogen (N) and phosphorus (P) availabilities are likely of essential importance (Bragazza et al., 2012; Hill et al., 2014; Spohn, 2020; Schillereff et al., 2021). In the past half-century, an increasing trend of N deposition from ∼87 to 94 Tg N yr^–1^ and P deposition from ∼3 to 4 Tg P yr^–1^ was observed (Wang et al., 2017b; Ackerman et al., 2019). Although with large spatial heterogeneity, the enlarged imbalance between N and P stoichiometry from deposition has posed unprecedented threats to ecosystem integrity and functioning (Peñuelas et al., 2013; Du et al., 2020) by weakening the stoichiometric homeostasis of organisms (Wang et al., 2016).

Accumulating evidence has shown that elevated N deposition exerted detrimental effects on peatland C sinks (Bubier et al., 2007; Limpens et al., 2011; Bragazza et al., 2012; Sheppard et al., 2014) with much less emphasis on P, despite its essential role in regulating peatland functioning (Larmola et al., 2013; Hill et al., 2014; Wang et al., 2016; Li et al., 2019). For example, at Mer Bleue bog in southeastern Canada, the combination of N, P, and potassium addition for 7–12 years showed much stronger effects on shrub production, peat decomposition (Larmola et al., 2013), and plant stoichiometry (Wang et al., 2016) than N-only addition. Moreover, in a peatland complex in northeastern China, the addition of P for 10 years modulated the effect of N on extracellular enzyme activity and organic matter decomposition (Li et al., 2019). Long-term addition of N and P could change plant community composition and exert a cascading effect (Ward et al., 2015; Carrara et al., 2018) on soil microbial community, in addition to changes in soil chemistry. For example, a shift in the composition of plant functional type (PFT) could alter the input of litter shedding from distinct PFTs with very different litter quality (Dorrepaal et al., 2005; Moore et al., 2008; Ward et al., 2015). The leached soluble phenolic compounds from litters could retard the metabolic activities of soil microorganisms, which are imperative in modulating peatland C stocks (Zak et al., 2019; Fenner and Freeman, 2020; Cory et al., 2022).

Although the large quantity of recalcitrant polyphenolics in litters from vascular plants has been considered the primary constraint on peat decomposition (e.g., Bragazza et al., 2013; Wang et al., 2015; Fenner and Freeman, 2020), emerging evidence has confirmed the potential of Sphagnum acid in regulating microbial activity and soil organic C stabilization in a peatland (Zhao et al., 2021). Therefore, it remains uncertain with regard to the regulatory mechanism of a shift in PFT abundance and composition, especially the expansion of shrubs at the expense of *Sphagnum* mosses, on soil microbial community structure in peatlands.

Soil microbial communities are vital players in terrestrial biogeochemical cycles, which significantly contribute to shaping the functioning of terrestrial ecosystems (Bardgett and van der Putten, 2014; Wagg et al., 2019) and are amenable to climate change (Cavicchioli et al., 2019; Jansson and Hofmockel, 2020). Although peatlands are acknowledged to contain large microbial populations with wide functional diversity (Gilbert and Mitchell, 2006; Thormann and Rice, 2007; Rydin and Jeglum, 2013), the waterlogged and cold soil conditions usually inhibit microbial activity and suppress their diversity (Andersen et al., 2013; Rydin and Jeglum, 2013), and hence contribute to C accumulation in peatlands.

It is well known that fungi-dominated ecosystems, especially *via* mycorrhizal associations, may accumulate more soil C than bacteria-dominated ecosystems because fungi often produce recalcitrant compounds, complex N-degrading enzymes, and have a higher C use efficiency (Lindahl et al., 2010; Jackson et al., 2017). Although there has been some debate that bacteria could be more active than fungi in northern peatlands owing to their superior resilience in response to environmental changes (e.g., Winsborough and Basiliko, 2010; Bissett et al., 2013), it is generally believed that fungi could establish competitive advantages over bacteria and archaea in bogs and poor fens given their broad enzymatic capacity and stress-tolerant growing strategy (Thormann, 2006; Golovchenko et al., 2007; Myers et al., 2012). As for those critical environmental constraints on soil microorganisms in northern peatlands, for example, anoxia, low pH, temperature, and nutrient availabilities (Andersen et al., 2013; Juan-Ovejero et al., 2020), we have not well characterized the complex effect of long-term nutrient enrichment on microbial community structure, especially *via* the cascading effect after changes in plant production and PFT composition.

After the loss of the “natural N filter” function of *Sphagnum* mosses when cumulative N deposition exceeds their immobilizing capacity, vascular plants may take advantage of increased available N in the soil (Lamers et al., 2000; Chiwa et al., 2016; Wieder et al., 2020). The expansion of vascular plants to the detriment of *Sphagnum* may favor fungal functional guilds associated with shrubs, trees, and graminoids, especially mycorrhizal fungi, but is likely detrimental to fungal guilds associated with *Sphagnum via* endophytic or biotrophic relationships (Kostka et al., 2016; Noordeloos et al., 2017; Borg Dahl et al., 2020). Besides, plant competition between PFT for nutrients will likely reshape the community of ericoid mycorrhizal (ErMF), arbuscular mycorrhizal (AMF), and ectomycorrhizal fungi (EcMF), which in turn regulates plant–plant interactions and ecosystem processes (Smith and Read, 2008; Tedersoo et al., 2020). Furthermore, cumulative N deposition may eventually weaken the symbiotic association of mycorrhizal fungi with their host plants, and increase C loss by facilitating the activity of saprotrophic fungi (Fernandez and Kennedy, 2016; Verbruggen et al., 2017; Vesala et al., 2021). In addition, we know little of what effects P addition and its interaction with N have on peatlands, which hinders our perception of important changes in the community structure of fungal functional guilds and implications on C storage in the face of uncertain N and P deposition regimes.

In this study, we established a pair of short- and long-term fertilization experiments in the ombrotrophic section of a peatland complex to assess how N and P additions influence fungal community structure. Considering that both short- and long-term N and/or P additions will relieve nutrient limitation, we hypothesized that, (H1) addition of N would shift the overall fungal community composition and diversity, with the effect being more profound when P is also added. Next, relative to 10 years of N and/or P additions, we expected the effect of 2 years of N and/or P additions on PFT abundance to be transient and minimal. Therefore, we hypothesized that, (H2) the cascading effect of altered PFT abundance on fungal community structure would be intense after long-term N and/or P additions. Furthermore, as nutrient enrichment will reduce plant dependence on mycorrhizal fungi to access limiting nutrients (Smith and Read, 2008; Leff et al., 2015; Vesala et al., 2021), we specifically hypothesized that, (H3) nutrient addition would decrease the relative abundance of mycorrhizal fungi and increase that of saprotrophs in general, and lignocellulose-degrading fungi (i.e., white-rot fungi) in particular. Given that *Sphagnum* are vulnerable to excess N addition, P addition may ameliorate this negative effect (Limpens et al., 2004; Carfrae et al., 2007). We further hypothesized that, (H4) the decline in *Sphagnum* cover after long-term N addition would decrease the relative abundance of *Sphagnum*-associated fungi, but P addition could alleviate its negative effect.

## Materials and Methods

### Study Site

Peatlands in China are mainly distributed in the northeastern and southwestern regions of the country, with a total area of ∼10,441 km^2^ and C storage of 1.39 Gt (Ma et al., 2012). This study was conducted at Hani Peatland, a bog–fen complex, (42°13′5′′ N, 126°31′05′′ E, 900 m above sea level) in the Changbai Mountains, northeastern China, with an area of 1,678 ha. Hani Peatland is located in the cold temperate zone with the continental monsoon climate. The mean annual precipitation is 757–930 mm and the mean annual temperature is 2.5–3.6°C (Bu et al., 2011). The dominant shrubs are *Betula fruticosa* Pall. var. *ruprechtiana* Trautv., *Vaccinium uliginosum* L., and *Rhododendron tomentosum* Harmaja. The dominant herbaceous plants are *Carex lasiocarpa* Ehrh., *Eriophorum polystachion* L., and *Smilacina japonica* A. Gray. The underlying moss layer is dominated by *Sphagnum palustre* L., *S. magellanicum* Bird., *S. fuscum* (Shimp.) Klinggr., and *S. capillifolium* (Ehrh.) Hedw. (Bu et al., 2017).

### Experimental Design

The short- (since 2018) and long-term (since 2007) N and P fertilization experiments were both established on large hummocks in the open region of Hani Peatland (Supplementary Figure 1). According to previous studies (Gunnarsson and Rydin, 2000; Güsewell et al., 2003), we used a full factorial design with three levels of N and three levels of P (Supplementary Table 1). The level of N addition was assumed to represent the range of 5–10 times of atmospheric deposition in this region (∼1 g m^–2^ yr^–1^). The N:P ratios from 5 to 20 were chosen to represent a rational range of N:P stoichiometry, resulting in the P addition level of 0.5 and 1 g m^–2^ yr^–1^. There were nine fertilization treatments with four completely randomized blocks and a total of 36 plots for each set of experiments. The size of each plot was 120 cm × 120 cm and 80 cm × 80 cm for the short- and long-term experiments, respectively. Ammonium nitrate (i.e., N fertilizer) and sodium dihydrogen phosphate (P fertilizer) were dissolved in distilled water and applied once a month from May to September (five times a year). The control plots were only supplied with distilled water.

### Soil Sampling

Peat samples from the long- and short-term fertilization plots were collected in August 2017 and 2019, respectively. Samples were taken when there was no precipitation for at least 10 days after fertilization. Living vegetation was removed before sampling and peat samples at 0–5 cm were randomly sampled in small patches to minimize the impact of destructive sampling. All peat samples were transported back to the laboratory on dry ice, homogenized, and divided into two subsamples. One subsample was stored at 4°C for physicochemical analysis within 2 weeks and the other subsample was stored at –80°C for soil DNA extraction and high-throughput sequencing.

### Vegetation Survey

Vegetation structure and species composition were estimated using the point intercept method (Larmola et al., 2013). Briefly, a customized stainless-steel frame (60 cm × 60 cm) with 61 grid points, was used for the measurement. The number of times (i.e., “hits”) a carbon fiber rod (2 mm in diameter) contacted the leaf (or leaves, if there were multiple leaves “hitting” the rod at different heights) of a vascular species or the capitulum of a *Sphagnum* species at each grid point was recorded. *Sphagnum* cover was calculated by the number of hits divided by 61 (i.e., the total number of grids). Vascular species abundance (not identical to species cover) was approximated by the total number of hits per unit area (i.e., hits m^–2^) for each species. The total abundance of a specific PFT was calculated by the sum of the abundance of all plant species within that PFT.

### DNA Extraction, Amplicon Generation, and Sequencing

A subsample of frozen peat (–80°C) was used for DNA extraction. Briefly, total DNA was extracted from 0.5 g peat using the PowerSoil DNA Isolation Kit (MoBio Laboratories Inc., Carlsbad, CA, United States) according to the manufacturer’s instructions. DNA was purified using the MoBio PowerClean DNA Clean-up Kit (MoBio Laboratories Inc., Carlsbad, CA, United States) and quantified with a Qubit Fluorometer (Invitrogen, Life Technologies, Carlsbad, CA, United States). Extracted DNA was then sent to Novogene (Novogen Co. Ltd., Beijing, China), where amplicon generation and sequencing proceeded as follows. The primers of ITS3-2024F (GCATCGATGAAGAACGCAGC) and ITS4-2490R (TCCTCCGCTTATTGATATGC) (White et al., 1990) were used to target the fungal ITS2 region. Full-length primers also included Illumina sequencing adaptors and a 16-bp index (on the reverse primer), which was unique to each sample. Samples from short- and long-term fertilized plots were sequenced on Illumina NovaSeq 6000 and HiSeq 2500 platforms (Illumina, Inc., San Diego, CA, United States) respectively, with 2 × 250 bp pair-end chemistry.

### Bioinformatics Analyses

Raw pair-end reads were demultiplexed by their unique barcodes using QIIME 1.9.1 (Caporaso et al., 2010). Primers were removed using Cutadapt (Martin, 2011) and pair-end reads were merged with BBmerge (Bushnell et al., 2017). Quality filtering was conducted using USEARCH (Edgar and Flyvbjerg, 2015) to discard low-quality reads based on three criteria: (1) expected error (estimated by error probabilities from Phred scores) > 1; (2) there were N bases; and (3) sequences length < 250-bp. Reference-based chimera filtering was conducted with UCHIME2 (Edgar et al., 2011) against the UNITE database (Version 28.06.2017) (Nilsson et al., 2015). The UPARSE pipeline (Edgar, 2013) was used to assign reads to operational taxonomic units (OTUs) at 97% similarity with singletons removed. The most abundant sequence in each OTU was selected as the representative sequence for further taxonomic annotation. Fungal taxonomic assignment was conducted with the BLAST algorithm and UNITE database (Version 20.02.2020) using QIIME. We further conducted BLASTn searches against the NCBI nucleotide database for OTUs within the top 500 (relative abundance) that were classified as non-fungal, no BLAST hit, or only assigned to a fungal class or higher. We eventually retained those dubious OTUs only if their BLASTn hits had a percent identity match of at least 75%, the coverage of at least 50%, and there was no better match with non-fungal organisms. The putative functional guilds of fungi were assigned using FUNGuild (Nguyen et al., 2016) and FungalTraits (Põlme et al., 2020), and we refined the assignments based on literature searches and our expertise in the unique ecology of peatlands (Wang et al., 2019). OTU tables were rarefied using a standard sequence number corresponding to the sample with the lowest sequencing depth (32,616 and 26,871 reads for short- and long-term fertilization, respectively). Subsequent analyses were all based on rarefied OTU tables.

### Statistical Analyses

All statistical analyses were run separately for two sets of fertilization experiments. Differences between N and/or P additions in OTU richness, Shannon’s diversity index, Pielou’s evenness index (i.e., Shannon’s diversity index divided by log-transformed OTU richness), PFT abundance, and key functional guilds of fungi (ErMF, EcMF, AMF, saprotrophs, lignocellulose-degrading fungi, and *Sphagnum*-associated fungi) were examined using linear mixed models in R 4.1.2 (R Core Team, 2021) with the *lmerTest* package (Kuznetsova et al., 2017). Linear mixed models included N, P addition, and their interaction as fixed factors, and block as a random factor. Models were fitted with Satterthwaite approximation for *F*-test. If a singular fit of a linear mixed model appeared, a linear model without random effect was used. If the main or interactive effect was significant, *emmeans* package (Lenth, 2021) was used to deprive marginal means and carry out *post hoc* pairwise comparisons. If the interactive effect was not statistically significant, only the main effect (i.e., N or P addition) would be examined and shown in figures and tables. Canonical analysis of principal coordinates (CAP) (Anderson and Willis, 2003) with Bray–Curtis dissimilarity was performed to visualize the overall pattern in OTU composition using PRIMER 7.0.21 (PRIMER-e, Quest Research Limited, Auckland, New Zealand). Permutational analysis of variance (PerMANOVA) with Bray–Curtis dissimilarity was conducted to assess the effect of N and/or P additions on OTU composition, followed by a test for homogeneity of multivariate dispersions (PermDISP). PerMANOVA model included N, P, and their interaction as fixed factors, and individual block as a random factor using PRIMER 7.0.21. Before PerMANOVA, OTU matrices were fourth root transformed to downweight the influence of the most abundant taxa. In addition, to identify OTUs with specific preferences to certain fertilization treatments, indicator species analysis was conducted using *indicspecies* package (De Cáceres and Legendre, 2009) in R.

Distance-based redundancy analysis (dbRDA) was used to evaluate the composition of fungal communities constrained by environmental variables using the *vegan* package (Oksanen et al., 2020) in R. The hierarchical partitioning method was used to assess the contribution of environmental variables to fungal community composition with the *rdacca.hp* package (Lai et al., 2022) in R.

## Results

**FIGURE 1:**
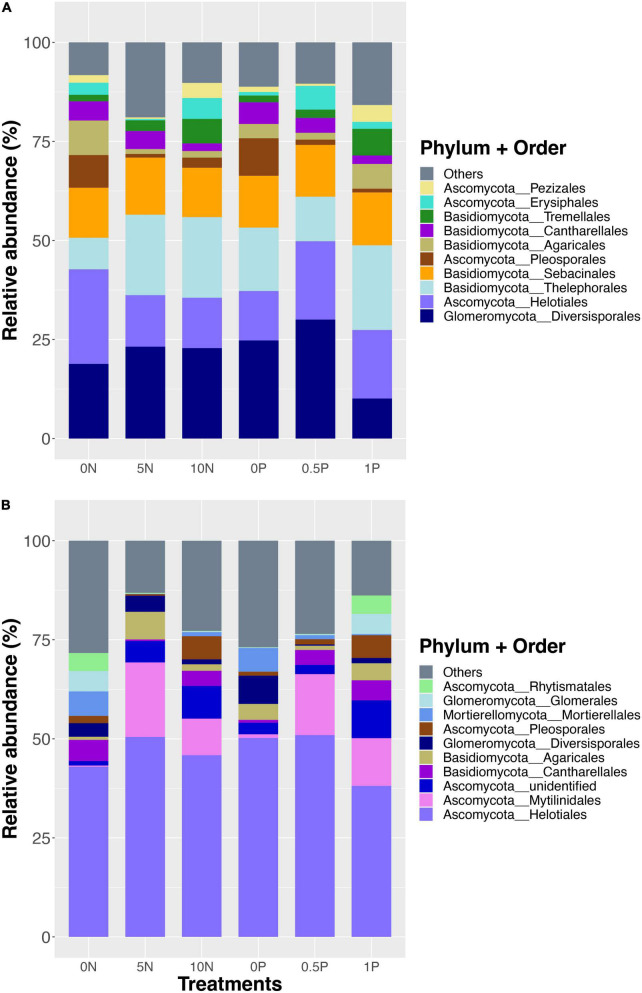
Relative abundance of dominant fungal orders after short- **(A)** and long-term **(B)** fertilization. Stacked bars are ordered by decreasing the number of total sequences per order (bottom to top) for each fertilization experiment separately. Treatment abbreviations as described in “Materials and Methods” section.

A total of 1,532,348 sequences from 36 samples were recovered and clustered into 770 OTUs in the short-term experiment, and the overall fungal community was dominated by Diversisporales (Glomeromycota), followed by Helotiales (Ascomycota), Thelephorales (Basidiomycota), Sebacinales (Basidiomycota), and Pleosporales (Ascomycota) (Figure 1A). For the long-term experiment, 2,103,347 sequences were recovered and clustered into 1,266 OTUs. Overall, the fungal community was dominated by Helotiales (Ascomycota), followed by Mytilinidales (Ascomycota), Cantharellales (Basidiomycota), Agaricales (Basidiomycota), and Diversisporales (Glomeromycota) (Figure 1B).

### Fungal Community Composition and Diversity

**FIGURE 2:**
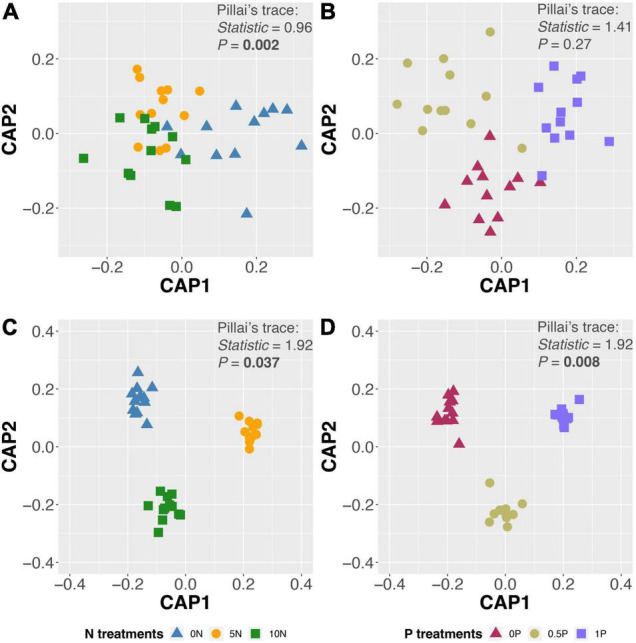
Canonical analysis of principal coordinates (CAP) ordinations with fungal operational taxonomic unit (OTU) composition after short-term N **(A)** and P **(B)** additions, and long-term N **(C)** and P **(D)** additions. Treatment abbreviations as described in “Materials and Methods” section.

Different addition rates and cumulative amounts of N or P addition exerted diverse effects on fungal community composition (Table 1). CAP ordinations and PerMANOVA revealed a significant effect of N addition on OTU composition (Figures 2A,C and Table 1); however, P addition only showed a significant impact on OTU composition in the long-term experiment, with no evidence of N by P interaction (Figures 2B,D and Table 1).

**TABLE 1:** PerMANOVA results of fungal community composition in response to different N and/or P addition treatments.

Analysis*	N treatments (*df F P*)	P treatments (*df F P*)	N × P treatments (*df F P*)	Block (*df F P*)
**Short-term experiment**				
PerMANOVA mixed model	2,24 1.54 **0.003**	2,24 1.17 0.15	4,24 0.91 0.77	3,24 3.72 **0.0001**
PermDISP	2,33 0.008 0.99	2,33 0.55 0.64	8,27 0.31 0.99	3,32 10.02 **0.001**
**Long-term experiment**				
PerMANOVA mixed model	2,24 1.41 **0.02**	2,24 1.77 **0.002**	4,24 1.06 0.29	3,24 1.61 **0.001**
PermDISP	2,33 0.06 0.95	2,33 0.002 0.99	8,27 0.54 0.91	3,32 5.89 **0.007**

P-values of significant models are highlighted in bold. *PerMANOVA mixed model include N treatment, P treatment and N × P treatments as fixed factors, and block as a random effect, to examine different community compositions among fixed factors. PermDISP is to examine differences in dispersion among N addition levels, P addition levels or all unique combination of N × P addition levels.

For the short-term experiment, fungal indicator OTUs of the 0N treatment (with all P addition levels pooled) included plant pathogens, and fungal lineages with broad functions (i.e., fungal OTUs with multiple putative functions), while indicators of 5N treatment mainly included AMF and EcMF (Supplementary Table 2). As for P addition, the indicator of 0P treatment was AMF, while the indicator OTUs of 0.5P and 1P treatments were EcMF, animal pathogens, and saprotrophs (Supplementary Table 2). In contrast, after long-term N and/or P additions, indicator OTUs have undergone marked changes. Specifically, indicators of 5N and 10N treatments were predominantly composed of saprotrophs. With regard to P additions, fungal indicators were shifted from AMF, ErMF, saprotrophs, and plant pathogens under 0P treatment to EcMF, saprotrophs, and fungal lineages with broad functions under 0.5P and 1P treatments.

Two sets of fertilization experiments showed different effects on fungal diversity (Table 2). Specifically, short-term additions of N and/or P at a medium rate (i.e., 5N and 0.5P, respectively) significantly increased OTU richness compared to 0N and 0P, respectively, but had no significant effect on Shannon’s diversity and Pielou’s evenness. In contrast, a significant interaction was found in the long-term experiment, in which N addition increased OTU richness when P was not added (Table 2). In combination with the 0.5P treatment, the 5N treatment significantly decreased Shannon’s diversity and Pielou’s evenness compared to the 0N treatment. A similar pattern was observed in the 5N treatment, which in combination with the 0.5P treatment significantly reduced Shannon’s diversity and Pielou’s evenness relative to the 0P treatment.

**TABLE 2:** Alpha-diversity indices (mean ± SE, *n* = 4) under different N and/or P fertilization treatments for short- and long-term experiments.

N treatments	P treatments	OTU richness	Shannon’s diversity	Pielou’s evenness
**Short-term experiment**				
0N		191 ± 7*AB*	3.1 ± 0.1	0.60 ± 0.03
5N		206 ± 11*B*	3.3 ± 0.2	0.62 ± 0.04
10N		180 ± 11*A*	3.0 ± 0.3	0.57 ± 0.05
	0P	190 ± 9*ab*	3.2 ± 0.2	0.60 ± 0.04
	0.5P	207 ± 13*b*	3.3 ± 0.3	0.61 ± 0.05
	1P	180 ± 6*a*	3.0 ± 0.1	0.58 ± 0.03
**Long-term experiment**				
0N	0P	205 ± 25*A*	2.8 ± 0.3	0.52 ± 0.05
	0.5P	283 ± 35	3.8 ± 0.6*B*	0.67 ± 0.08*B*
	1P	274 ± 41	3.2 ± 0.4	0.56 ± 0.05
5N	0P	285 ± 29*AB*	3.8 ± 0.3*b*	0.68 ± 0.04*b*
	0.5P	210 ± 24	2.1 ± 0.6*A*,*a*	0.39 ± 0.11*A*,*a*
	1P	270 ± 19	2.9 ± 0.3*ab*	0.52 ± 0.05*ab*
10N	0P	294 ± 21*B*	3.3 ± 0.4	0.58 ± 0.06
	0.5P	246 ± 21	3.1 ± 0.5*AB*	0.57 ± 0.08*AB*
	1P	233 ± 17	2.9 ± 0.3	0.53 ± 0.05

*OTU, operational taxonomic units. The interaction between N and P treatment is not significant in the short-term experiment, therefore only the main effects are shown. Different uppercase letters represent significant differences (P < 0.05) among N treatments with all P addition levels pooled (short-term experiment), or among N treatments at the same P addition level (long-term experiment). Different lowercase letters represent significant differences (P < 0.05) among P treatments with all N addition levels pooled (short-term experiment), or among P treatments at the same N addition level (long-term experiment).*

### Response of Plant Functional Type Abundance

**FIGURE 3:**
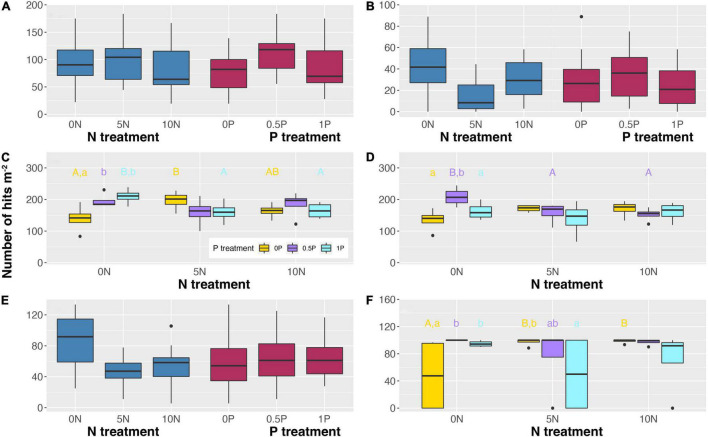
Abundance of ericaceous shrubs **(A)**, ectomycorrhizal plants **(B)**, arbuscular mycorrhizal plants **(C)**, graminoids **(D)**, deciduous shrubs **(E)**, and cover of *Sphagnum* **(F)** in response to short-term N and/or P additions. When the interactive effect is not significant **(A,B,E)**, only the main effects are shown. Different uppercase letters represent significant differences (*P* < 0.05) among N treatments under the same P addition level. Different lowercase letters represent significant differences (*P* < 0.05) among P treatments under the same N addition level. Treatment abbreviations as described in “Materials and Methods” section.

Different rates and cumulative amounts of N and/or P additions also exerted diverse effects on the abundance of different PFTs. Unexpectedly, even 2-year additions of N and/or P have exerted significant effects on PFT abundance (Figure 3). Specifically, a high rate of N addition (i.e., 10N) reduced the relative abundance of ericaceous shrubs by 15% (Figure 3A) while a medium rate of N addition (i.e., 5N) decreased that of ectomycorrhizal plants by 64% (Figure 3B). As for P addition, the 0.5P treatment increased them by 45 and 172%, respectively. In contrast, with a significant interactive effect, N addition increased the abundance of arbuscular mycorrhizal plants and *Sphagnum* by 17–41% and 102–105%, respectively, when P was not added (Figures 3C,F). However, it decreased the abundance of arbuscular mycorrhizal plants under 1P treatment by 22–24% (Figure 3C) and that of graminoids under 0.5P treatment by 24–27% (Figure 3D). Moreover, when N was not added, P addition increased the abundance of arbuscular mycorrhizal plants (by 40–50%) and *Sphagnum* cover (by 97–108%) at both medium and high rates (Figures 3C,F), and increased that of graminoids by 55% at a medium rate (Figure 3D). In contrast, in combination with the 5N treatment, P addition decreased *Sphagnum* cover by 23–49% (Figure 3F). Furthermore, N addition reduced the abundance of deciduous shrubs by 35–43% with no significant effect of P addition (Figure 3E).

**FIGURE 4:**
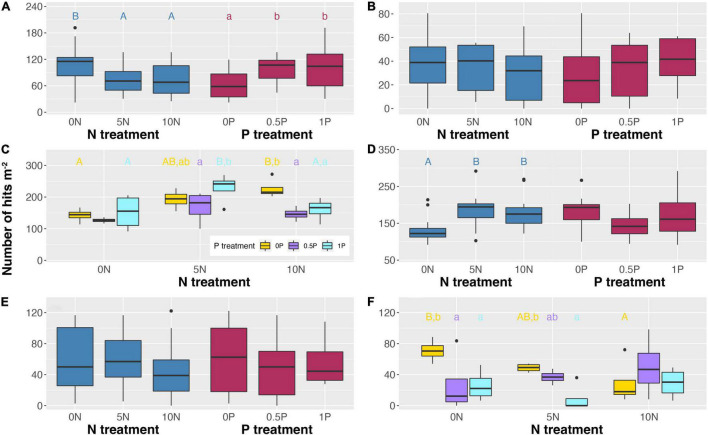
Abundance of ericaceous shrubs **(A)**, ectomycorrhizal plants **(B)**, arbuscular mycorrhizal plants **(C)**, graminoids **(D)**, deciduous shrubs **(E)**, and cover of *Sphagnum* **(F)** in response to long-term N and/or P additions. When the interactive effect is not significant **(A,B,D,E)**, only main effects are shown. Different uppercase letters represent significant differences (*P* < 0.05) among N treatments in **(A,D)**, and N treatments under the same P addition level in **(C,F)**. Different lowercase letters represent significant differences (*P* < 0.05) among P treatments in **(A)**, and P treatments under the same N addition level in **(C,F)**. Treatment abbreviations as described in “Materials and Methods” section.

As expected, 10-year additions of N and/or P showed significant effects on PFT abundance (Figure 4). Specifically, N addition decreased the abundance of ericaceous shrubs by ∼32% but increased that of graminoids by 36–40% (Figures 4A,D). Similar to the short-term experiment, the abundance of arbuscular mycorrhizal plants was increased by 36–59% after N addition when P was not added (Figure 4C). However, contrasting effects were often observed between two sets of experiments. Specifically, long-term N addition increased the abundance of arbuscular mycorrhizal plants by 50% at a medium rate in combination with the 1P treatment (Figure 4C) but decreased *Sphagnum* cover by 31–59% when P was not added (Figure 4F). As for P addition, it increased the abundance of ericaceous shrubs and ectomycorrhizal plants by 61–65% and 14–347%, respectively (Figures 4A,B). Furthermore, P addition increased the abundance of arbuscular mycorrhizal plants by 18% in combination with the 5N treatment but decreased it by 29–35% in combination with the 10N treatment (Figure 4C), and decreased *Sphagnum* cover by 24–82% in combination with the 0N and 5N treatments (Figure 4F).

### Response of Fungal Functional Guilds

**FIGURE 5:**
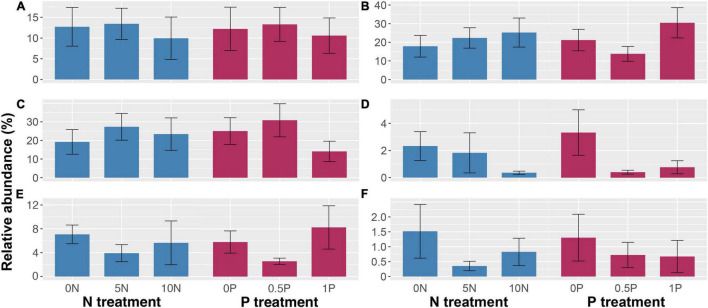
Relative abundance (mean ± SE) of ericoid mycorrhizal fungi **(A)**, ectomycorrhizal fungi **(B)**, arbuscular mycorrhizal fungi **(C)**, lignocellulose-degrading fungi **(D)**, saprotrophic fungi (not including lignocellulose-degrading fungi) **(E)**, and *Sphagnum*-associated fungi **(F)** in response to short-term N and/or P additions. Treatment abbreviations as described in “Materials and Methods” section.

Given the very large spatial heterogeneity, hypervariable community composition, and relatively small sample size, we did not obtain any statistically significant result with regard to the responses of different fungal functional guilds to fertilization. However, some clear but complex patterns were observed. Generally, short-term N and/or P additions did not remarkably reduce the relative abundances of ErMF, EcMF, and AMF (Figures 5A–C), except for AMF, which was decreased by 44% under the 1P treatment. In contrast, N and P additions decreased the relative abundance of lignocellulose-degrading fungi by 22–83% and 76–88%, respectively (Figure 5D), and decreased that of *Sphagnum*-associated fungi by 45–77% and 45–48%, respectively (Figure 5F). Unexpectedly, P addition decreased the relative abundance of saprotrophs by 55% under the 0.5P treatment (Figure 5E). There was a negative correlation between the relative abundance of AMF and saprotrophs (Spearman’s ρ = –0.32, *P* = 0.058).

**FIGURE 6:**
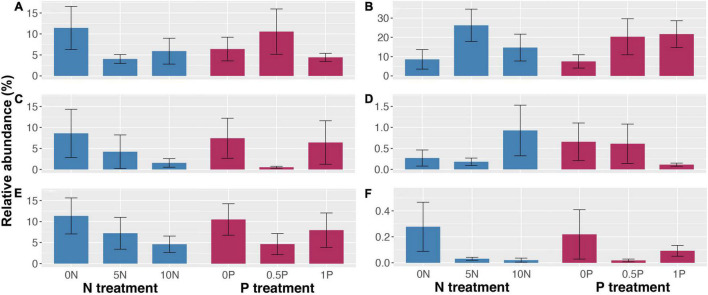
Relative abundance (mean ± SE) of ericoid mycorrhizal fungi **(A)**, ectomycorrhizal fungi **(B)**, arbuscular mycorrhizal fungi **(C)**, lignocellulose-degrading fungi **(D)**, saprotrophic fungi (not including lignocellulose-degrading fungi) **(E)**, and *Sphagnum*-associated fungi **(F)** in response to long-term N and/or P additions. Treatment abbreviations as described in “Materials and Methods” section.

Long-term N addition decreased the relative abundance of ErMF and AMF by 48–65% and 51–81%, respectively (Figures 6A,C), but increased that of EcMF by 71–205% (Figure 6B). However, the effect of P addition on the relative abundance of mycorrhizal fungi was complex: it increased the relative abundance of EcMF by 171–188% (Figure 6B), and increased that of ErMF by 66% but decreased that of AMF by 93% under 0.5P treatment (Figures 6A,C). In addition, long-term N addition doubled the relative abundance of lignocellulose-degrading fungi but decreased that of saprotrophs by 36–59% (Figures 6D,E). The relative abundance of lignocellulose-degrading fungi was reduced by 77% under 1P treatment (Figure 6D), and a 55 and 24% decline in the relative abundance of saprotrophs was observed under the 0.5P and the 1P treatments, respectively (Figure 6E). Unlike the short-term effect, there were negative correlations between the relative abundance of saprotrophs and ErMF (Spearman’s ρ = –0.30, *P* = 0.075) and EcMF (Spearman’s ρ = –0.35, *P* = 0.035) with regard to the long-term effect. Like the short-term experiment, the relative abundance of *Sphagnum*-associated fungi was reduced by 88–93% and 59–91% in response to long-term N and P additions, respectively (Figure 6F).

### Effect of Plant Functional Type Abundance and Environmental Factors

Overall, TN concentration and vegetation abundance (evergreen and deciduous shrubs, and *Sphagnum*) accounted for 68% of the total variation in OTU composition (10% adjusted) in the short-term experiment (Supplementary Table 3). In contrast, peat chemistry (TC concentration and humification indices) and the abundance of deciduous shrubs accounted for 64% of total variation (11% adjusted) in the long-term experiment.

In general, slightly stronger correlations between the relative abundance of different fungal functional guilds and PFT abundance appeared in the long-term experiment compared to the short-term one (Table 3). Unexpectedly, we did not anticipate that there were only a few significant correlations between the relative abundance of mycorrhizal fungi and their host plants (Table 3). Notably, only the relative abundance of ErMF in the short-term experiment and EcMF in the long-term experiment showed significantly positive correlations with the abundance of ericaceous shrubs and ectomycorrhizal plants, respectively (Table 3). In addition, there was no significant correlation between the relative abundance of *Sphagnum*-associated fungi and *Sphagnum* cover in neither experiment (Table 3).

**TABLE 3:** Spearman’s correlation between the relative abundance of different fungal functional guilds and abundance of different plant functional types.

	Ericaceous shrubs	EcM plants	AM plants	Graminoids	Deciduous shrubs	*Sphagnum* spp.
**Short-term experiment**						
ErMF	**0.56** (0.0004)	–0.22 (0.199)	**0.50** (0.002)	0.17 (0.331)	–0.04 (0.818)	0.19 (0.273)
EcMF	–0.32 (0.056)	0.16 (0.352)	0.04 (0.834)	0.08 (0.662)	0.12 (0.483)	–0.16 (0.339)
AMF	–0.10 (0.554)	–0.21 (0.214)	–0.19 (0.278)	0.01 (0.94)	–**0.34** (0.042)	0.26 (0.129)
Ligno	–0.05 (0.763)	0.08 (0.657)	0.13 (0.444)	–0.08 (0.640)	0.04 (0.800)	–0.22 (0.206)
Sapro	0.03 (0.856)	**0.36** (0.029)	–0.21 (0.209)	–**0.39** (0.020)	0.22 (0.197)	–0.11 (0.506)
Sphag	0.11 (0.517)	–0.07 (0.698)	0.32 (0.054)	–0.01 (0.963)	0.26 (0.119)	0.04 (0.838)
**Long-term experiment**						
ErMF	0.16 (0.352)	0.15 (0.373)	–**0.33** (0.050)	–**0.44** (0.007)	0.18 (0.283)	–0.12 (0.479)
EcMF	–0.26 (0.125)	**0.44** (0.007)	0.27 (0.109)	0.15 (0.388)	**0.41** (0.013)	–**0.44** (0.017)
AMF	0.06 (0.747)	–0.21 (0.216)	–0.23 (0.171)	–0.15 (0.368)	–0.27 (0.109)	**0.41** (0.012)
Ligno	0.16 (0.352)	–0.03 (0.855)	–0.02 (0.912)	–0.17 (0.324)	0.18 (0.289)	0.16 (0.363)
Sapro	0.05 (0.776)	–0.30 (0.078)	–0.12 (0.502)	0.005 (0.979)	–**0.35** (0.037)	0.30 (0.074)
Sphag	0.05 (0.794)	0.14 (0.401)	0.06 (0.716)	–0.12 (0.471)	**0.40** (0.026)	0.11 (0.515)

*Spearman’s rank correlation coefficients are shown with P-values in parentheses. Significant (P < 0.05) coefficients are highlighted in bold. ErMF, ericoid mycorrhizal fungi; EcMF, ectomycorrhizal fungi; AMF, arbuscular mycorrhizal fungi; Ligno, lignocellulose-degrading fungi; Sapro, saprotrophic fungi (not including lignocellulose-degrading fungi); Sphag, Sphagnum-associated fungi; Ericaceous shrubs, ericoid mycorrhizal plants; EcM plants, ectomycorrhizal plants; AM plants, arbuscular mycorrhizal plants.*

Being a notable proportion of saprotrophs (6–57%), the relative abundance of lignocellulose-degrading fungi showed a significant correlation with that of saprotrophs in the short-term experiment (Spearman’s ρ = 0.33, *P* = 0.045). However, with a sharp decline in the relative abundance of lignocellulose-degrading fungi, we did not observe such a correlation in the long-term experiment (Spearman’s ρ = 0.14, *P* = 0.407).

There were only very few significant correlations between the relative abundance of different fungal functional guilds and environmental factors (Supplementary Table 4). Notably, the relative abundance of EcMF in both experiments showed significant correlations with different humification indices.

## Discussion

### Strong Effects of N and/or P Additions on Fungal Community Composition and Diversity

Partially supporting our first hypothesis, both short- and long-term N additions significantly changed fungal community composition and diversity, but the effect was not modulated by P addition, nor was there a significant interaction (Table 1). The effect of accumulative N and/or P additions over a long period seems to override the effect of addition rate on peatland ecosystem functioning (e.g., Larmola et al., 2013). As excepted, the long-term addition of N or P exerted a strong impact on the fungal community (Figure 1), and similar results were demonstrated in other studies (e.g., Carrara et al., 2018; Kiheri et al., 2020; Vesala et al., 2021). A clear shift in community composition appears from being shared by copiotrophic Ascomycota, and oligotrophic Basidiomycota, and Glomeromycota (Yao et al., 2017) after short-term N and/or P additions, to being overwhelmingly dominated by copiotrophic Ascomycota after N and/or P additions over 10 years.

A medium rate of N or P addition increased OTU richness in the short-term experiment, but significantly reduced Shannon’s diversity and Pielou’s evenness when added in combination in the long-term experiment (Table 2). This increased OTU richness was accompanied by decreases in the relative abundance of EcMF (e.g., Thelephorales) and saprotrophs, and an increase in that of AMF (e.g., Diversisporales) (Figures 1A, 5B,C,E), indicating the changes in overall fungal community structure is closely linked to the shift in the relative abundance of mycorrhizal fungi. Our results are in line with the notion that mycorrhizal fungi are of great importance in peatlands (Thormann, 2006; Andersen et al., 2013; Juan-Ovejero et al., 2020). Furthermore, a decline in Shannon’s diversity in the long-term experiment is driven by the changes in Pielou’s evenness (Table 2), suggesting the OTU distribution pattern is shifted into stronger clumped dispersion rather than greater taxonomic richness (Taylor, 2002; Tan et al., 2021).

### The Weak Cascading Effect of a Shift in Plant Functional Type on Fungal Community Structure

Although PFT abundance changed substantially after N and/or P additions, the anticipated cascading effect on the fungal community was weak, which is inconsistent with our second hypothesis. This may be because our surface peat cannot capture the complex vertical stratification of the fungal community associated with vascular plants with different rooting depths in peatlands (e.g., Lin et al., 2014; Lamit et al., 2021). Unexpectedly, even 2 years of N and/or P additions also significantly changed PFT abundance, but the effects were complex. For example, a high rate of N addition was detrimental to ericaceous shrubs, but whether the effect was positive or negative was dependent on the P addition rate for arbuscular mycorrhizal plants and *Sphagnum*, owing to significant N by P interaction (Figure 3). Without concomitant addition of P, the addition of N increased the abundance of both arbuscular mycorrhizal plants and *Sphagnum*, indicating the accelerated growth of vascular plants after N enrichment is not necessarily at the expense of *Sphagnum* from a short-term perspective. Our finding does not support the contention that vascular plants will only have access to enriched nutrients after the loss of the “natural N filter” function of *Sphagnum* in response to excess N input (Lamers et al., 2000; Chiwa et al., 2016).

With regard to long-term effects, a negative impact of N on the abundance of ericaceous shrubs became more intensive while ectomycorrhizal plants no longer benefited from a high rate of N addition (Figures 4A,B). Unlike the short-term effect, 10 years of cumulative addition of N was of benefit to arbuscular mycorrhizal plants but decreased *Sphagnum* cover (Figures 6C,F). The declined *Sphagnum* cover may compromise the growth of ericaceous shrubs because Ericaceae roots heavily in the upper moss layer (Read et al., 2004). Furthermore, a loss of living *Sphagnum* will lead to the subsidence of bare peat and the subsequent saturated conditions are not favorable for the growth of ericaceous shrubs. The significant correlation between the abundance of ericaceous shrubs and the relative abundance of ErMF is in line with the finding that the removal of Ericaceae can cause a remarkable decrease in ErMF abundance (Lamit et al., 2021).

Furthermore, P addition increased the abundance of ericaceous shrubs and ectomycorrhizal plants but decreased *Sphagnum* cover (Figures 4A,B,F), suggesting that P addition does not alleviate the negative impact of N addition on *Sphagnum* in peatlands (Fritz et al., 2012). We strongly recommend that the critical load derived from the cumulative amount of N and/or P additions would be a more appropriate property than the rate (e.g., Lamers et al., 2000; Bragazza et al., 2004; Wieder et al., 2020; Zhou et al., 2021) to assess the effect of nutrient enrichment on peatland ecosystem processes and functioning.

Overall, the abundances of different PFTs were important factors in explaining the variations in OTU composition, but the essential role of changing soil chemistry was mainly detected after long-term fertilization (Supplementary Table 3). We did not find close relationships between the relative abundance of fungal functional guilds and related PFTs, *albeit* we did find such relationships were slightly stronger in the long-term experiment (Table 3). Therefore, these results do not support our second hypothesis with regard to the anticipated cascading effect of changing PFT abundance on fungal community composition. Three possible reasons may explain these discrepancies. First, different species within a specific PFT may respond differently to N and/or P additions (i.e., intra-PFT variation). For example, with regard to the dominant Ericaceae in our site, the abundance of *Chamaedaphne calyculata* was decreased by N addition, whereas that of *Rhododendron tomentosum* and *Vaccinium uliginosum* varied depending on rates of N addition (Supplementary Figures 2,3). Whether there is a ubiquitous species-specific symbiosis between host plants and mycorrhizal fungi in response to nutrient enrichment in peatlands is largely unknown. Second, below-ground response to nutrient enrichment may be different from the above-ground compartment and this discrepancy may vary between different PFTs, which has been reported in a rich fen and an arctic tundra ecosystem (El-Kahloun et al., 2003; Wang et al., 2017a). For example, P had been elaborated to increase above-ground biomass while N tended to decrease live root biomass but increase dead root biomass (El-Kahloun et al., 2003). Therefore, the shift in PFT abundance we observed may not reflect the changes in below-ground biomass, productivity, or morphological characteristics (Lamit et al., 2021). Third, some mycorrhizal fungi have retained efficient saprotrophic capacity (Smith et al., 2017; Verbruggen et al., 2017; Martino et al., 2018), and may not function as symbionts as indicated by their taxonomic information. Therefore, it merits further investigation to disentangle multiple mechanisms that drive the relationship between vegetation and their housed fungal community.

### Relationship Between Mycorrhizal and Saprotrophic Fungi

Like the effects on overall fungal community structure and PFT abundance, short- and long-term fertilizations exerted contrasting influences on the relative abundance of different fungal functional guilds. AMF is often considered to be less favorable to their hosts after nutrient additions, therefore decreases in their relative abundances are usually reported (e.g., Johnson et al., 2010; Leff et al., 2015). However, our short-term N and/or P additions did not significantly decrease the relative abundance of mycorrhizal fungi to recruit more saprotrophs (Figure 5), which is contrary to our third hypothesis.

As for the long-term effect, we did find declines in the relative abundances of ErMF and AMF (but not EcMF) and a concomitant increase in the relative abundance of lignocellulose-degrading fungi after N addition, but no such pattern was observed for general saprotrophs (Figure 6), partially supporting our third hypothesis. These results are in line with those of Lekberg et al. (2021) but in contrast with Averill et al. (2018), which corroborated that N deposition favored AMF at the expense of EcMF because the former primarily relied on inorganic N sources while the latter relied more heavily on organic N sources *via* their powerful capacity to produce N-degrading enzymes. Besides, we noticed negative correlations between the relative abundance of AMF and saprotrophs in the short-term experiment, and between that of ErMF, EcMF, and saprotrophs in the long-term experiment, conditionally supporting the “Gadgil effect” (Gadgil and Gadgil, 1975; Fernandez and Kennedy, 2016). However, cautions must be taken not to overinterpret our findings as the correlations were functional guilds dependent and they varied with the duration of nutrient enrichment. It is also possible that this corresponds to a statistical artifact because some functional guilds will have a larger relative abundance than others and *vice versa*.

Because our study is based on the relative abundance of fungal functional guilds, we are not confident to declare the phenomena we observed are ubiquitous in other peatland ecosystems, but we do find some paralleled studies which have revealed comparable conclusions. For example, Kiheri et al. (2020) showed that 15 years of N and/or NPK additions did not reduce mycorrhizal colonization of ericaceous plant roots, likely owing to the alternative benefits of mycorrhizal fungi to host plants other than nutrient acquisition. In addition, Clemmensen et al. (2006) described soil fungal biomass and ErMF mycelial production both increased after 14 years of NPK addition in two arctic tundra ecosystems, which was partly caused by enhanced growth of ectomycorrhizal plants and partly caused by stimulation of fungal growth. However, at the global scale, N and P additions have been confirmed to reduce the relative abundance of mycorrhizal fungi in grassland ecosystems (Leff et al., 2015). Whether the responses of different fungal functional guilds to nutrient enrichment are ecosystem-specific will be an interesting topic for further assessment.

### *Sphagnum* and *Sphagnum*-Associated Fungi

Short- and long-term N additions exerted a contrasting effect on *Sphagnum* cover but consistently reduced the relative abundance of *Sphagnum*-associated fungi, which supports our fourth hypothesis with regard to the long-term negative effect of N on *Sphagnum* and associated fungi. *Sphagnum* cover was increased when N or P was added individually in the short-term experiment (Figure 3F), *albeit* the relative abundance of *Sphagnum*-associated fungi was decreased (Figure 5F), indicating that fungi are more susceptible to nutrient enrichment than *Sphagnum*, which has a certain degree of resilience (Turetsky et al., 2012; Chiwa et al., 2016; Wang et al., 2016).

*Clavaria sphagnicola*, *Galerina tibiicystis*, *G. sphagnicola*, and *G. paludosa* (Kostka et al., 2016; Borg Dahl et al., 2020) were recovered in this study and there were far fewer sequences in samples from the long-term experiment (1,289) relative to the short-term one (10,550). The lack of relationship between *Sphagnum* mosses and those fungi inhabiting their tissues and surface may be owing to the contrasting responses of different species. For example, the relative abundance of *C. sphagnicola* was increased by 2.5 times after N addition while that of *G. sphagnicola* was decreased by 97% (Supplementary Figure 4). Besides, the relative abundance of *C. sphagnicola* was increased by 32 times after P addition while that of *G. tibiicystis* was decreased by 99% (Supplementary Figure 5). However, our knowledge of species identity and its function is still in its infancy. *Sphagnum*-associated fungi have the potential to act as mutualists, symbionts, or antagonists of *Sphagnum* (Kostka et al., 2016), therefore it is imperative to investigate the mechanisms of *Sphagnum*–fungi interaction with targeted sequencing technique and the metabolic potential of the associated fungal population.

Unexpectedly, long-term addition of P also reduced *Sphagnum* cover as well as the relative abundance of *Sphagnum*-associated fungi instead of alleviating the negative effect of N addition, which is inconsistent with our fourth hypothesis. This result supports the notion that there is only a limited alleviating effect of P addition on excess N (Fritz et al., 2012; Chiwa et al., 2018), likely owing to imposed physiological stress (Fritz et al., 2012). Collectively, with regard to our fourth hypothesis, we did observe that long-term N addition decreased the relative abundance of *Sphagnum*-associated fungi, but P addition did not alleviate the negative effect of excess N input. Considering that the loss of *Sphagnum* is one of the most significant effects of nutrient additions in peatlands (Bubier et al., 2007; Limpens et al., 2011; Levy et al., 2019; Wieder et al., 2020), it is of paramount importance to delineate the relationship between *Sphagnum* and its inhabiting fungi to attain a predictive understanding of peatland ecosystem functioning in the face of global change.

## Conclusion

Our study did not find evidence to support most of our raised hypotheses with regard to the effect of different rates and cumulative amounts of N and/or P additions on fungal community composition and diversity, PFT abundance, as well as the relative abundances of several essential fungal functional guilds in a northern peatland. Overall, fungal community composition and diversity changed after N addition, but the effect was neither intensified nor alleviated after P addition. Unexpectedly, even 2-year nutrient addition can alter PFT abundance but there was not a strong cascading effect on the fungal community. Long-term nutrient addition showed a stronger effect on the relative abundance of fungal functional guilds, and we observed a weak “Gadgil effect” between mycorrhizal fungi and saprotrophs. Moreover, a decline in *Sphagnum* cover after long-term N addition was not accompanied by concomitant changes in *Sphagnum*-associated fungi, which was likely owing to different species-specific responses of *Sphagnum*-associated fungi to nutrient enrichment. Given the most contrasting results are disclosed between the two sets of experiments, our study highlights the necessity of assessing the long-term effect, or at least the cumulative effect, of nutrient enrichment on the association between vegetation and microbial community in peatland ecosystems. With large spatial heterogeneity and limited sample size, the ubiquity of our findings should be further validated. We propose that future research priorities should focus on the connection between microbial community structure and functionality.

## Data Availability Statement

The datasets presented in this study can be found in online repositories. The names of the repository/repositories and accession number(s) can be found below: https://www.ncbi.nlm.nih.gov/, PRJNA826221.

## Author Contributions

MW and Z-JB were involved in the experimental design. CC, JH, TL, and MW conducted the data analyses. CC, JH, LG, TL, ZL, SL, and MW performed the field sampling and laboratory analyses. All authors were involved in manuscript preparation.

## Conflict of Interest

The authors declare that the research was conducted in the absence of any commercial or financial relationships that could be construed as a potential conflict of interest.

## Publisher’s Note

All claims expressed in this article are solely those of the authors and do not necessarily represent those of their affiliated organizations, or those of the publisher, the editors and the reviewers. Any product that may be evaluated in this article, or claim that may be made by its manufacturer, is not guaranteed or endorsed by the publisher.
