# Supplementary material for: Does Shift in Vegetation Abundance After Nitrogen and Phosphorus Additions Play a Key Role in Regulating Fungal Community Structure in a Northern Peatland?
Source: Front Microbiol. 2022 Jun 9;13:920382. doi: 10.3389/fmicb.2022.920382 (PMC9224414; doi:10.3389/fmicb.2022.920382)
Supplement: Supplementary file 1 [file Table_1.docx]

**Supplementary Materials**

**Supplementary Methods**

***Soil physicochemical analysis***

Peat water content was measured by oven-drying at 65 °C to a constant weight. Dried peat was ground with a ball mill (MM400, Retsch GmbH, Haan, Germany) to determine the concentrations of total carbon (TC), total nitrogen (TN) and total phosphorus (TP). TN and TC were measured with dry combustion on an elemental analyzer (vario MACRO cube, elementar, Germany). TP was determined colorimetrically using the ammonium molybdate-ascorbic acid method (Murphy and Riley 1962) on a continuous flow analyzer (San++, Skalar Analytical, Breda, The Netherlands) with digested samples in concentrated sulphuric acid and hydrogen peroxide with selenium and lithium sulphate as catalysts (Parkinson and Allen 1975).

***Fourier-transform infrared (FTIR) spectroscopic analysis***

The organic chemistry of dried peat sample was measured on a FTIR spectrometer (Vetex70, BRUKER, Germany) using the FTIR spectroscopic analysis. Specifically, 2 mg of ground peat was mixed with 200 mg potassium bromide and homogenized with an Agate Mortar. Mixture was put into the pellet die and pressed at 6.5 tons load for 15 seconds. The range of spectra was 4000 to 400 cm^-1^ with a resolution of 4 cm^-1^. Sixteen scans were collected for each sample. The background of potassium bromide was removed from the spectra and then baseline corrected. To determine the degree of humification of peat sample, peaks around the following wavenumbers were recorded: 1090 cm^-1^ (polysaccharide), 1420 cm^-1^ (phenolic and aliphatic structures), 1510 cm^-1^ (amides), 1630 cm^-1^ (aromatics and aromatic or aliphatic carboxylates), and 1720 cm^-1^ (carboxylic acids and aromatic esters) (Niemeyer et al., 1992; Moore et al., 2019; Drollinger et al., 2020). Furthermore, humification indices were calculated as the ratios of corresponding peaks, i.e., 1420/1090, 1510/1090, 1630/1090, and 1720/1090 (Niemeyer et al., 1992; Cocozza et al., 2003; Broder et al., 2012), the decrease of which was considered to reflect the degree of enhanced organic matter decomposition (Niemeyer et al., 1992; Broder et al., 2012).

**References**

Broder, T., Blodau, C., Biester, H., and Knorr, K.H. (2012). Peat decomposition records in three pristine ombrotrophic bogs in southern Patagonia. *Biogeosciences* 9, 1479-1491. doi: 10.5194/bg-9-1479-2012

Cocozza, C., D'orazio, V., Miano, T.M., and Shotyk, W. (2003). Characterization of solid and aqueous phases of a peat bog profile using molecular fluorescence spectroscopy, ESR and FT-IR, and comparison with physical properties. *Org. Geochem.* 34, 49-60. doi: 10.1016/S0146-6380(02)00208-5

Drollinger, S., Knorr, K.-H., Knierzinger, W., and Glatzel, S. (2020). Peat decomposition proxies of Alpine bogs along a degradation gradient. *Geoderma* 369, 114331. doi: 10.1016/j.geoderma.2020.114331

Moore, T.R., Knorr, K.-H., Thompson, L., Roy, C., and Bubier, J.L. (2019). The effect of long-term fertilization on peat in an ombrotrophic bog. *Geoderma* 343, 176-186. doi: 10.1016/j.geoderma.2019.02.034

Niemeyer, J., Chen, Y., and Bollag, J.-M. (1992). Characterization of humic acids, composts, and peat by diffuse reflectance Fourier-transform infrared spectroscopy. *Soil Sci. Soc. Am. J.* 56, 135-140. doi: 10.2136/sssaj1992.03615995005600010021x

**Supplementary Figure S1.** A schematic map showing the locations of the plots for the short- and long-term experiments.

**
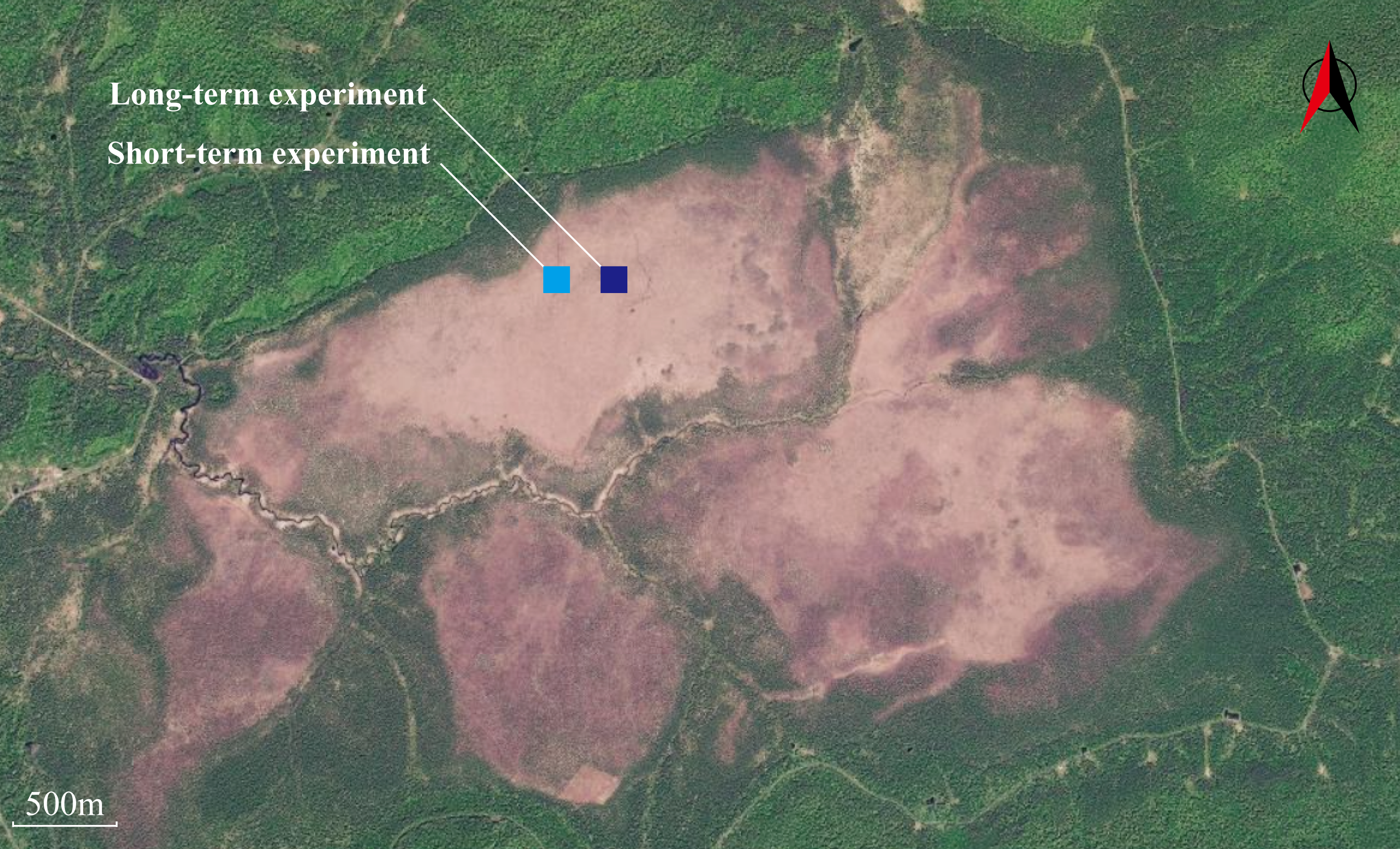
**

**Supplementary Figure S2** Abundance of *Chamaedaphne calyculata* (A), *Rhododendron tomentosum* (B) and *Vaccinium uliginosum* (C) in response to short-term N and/or P additions. Different lowercase letters represent significant differences (*P* < 0.05) among P treatments. Treatment abbreviations as described in Materials and Methods.

**
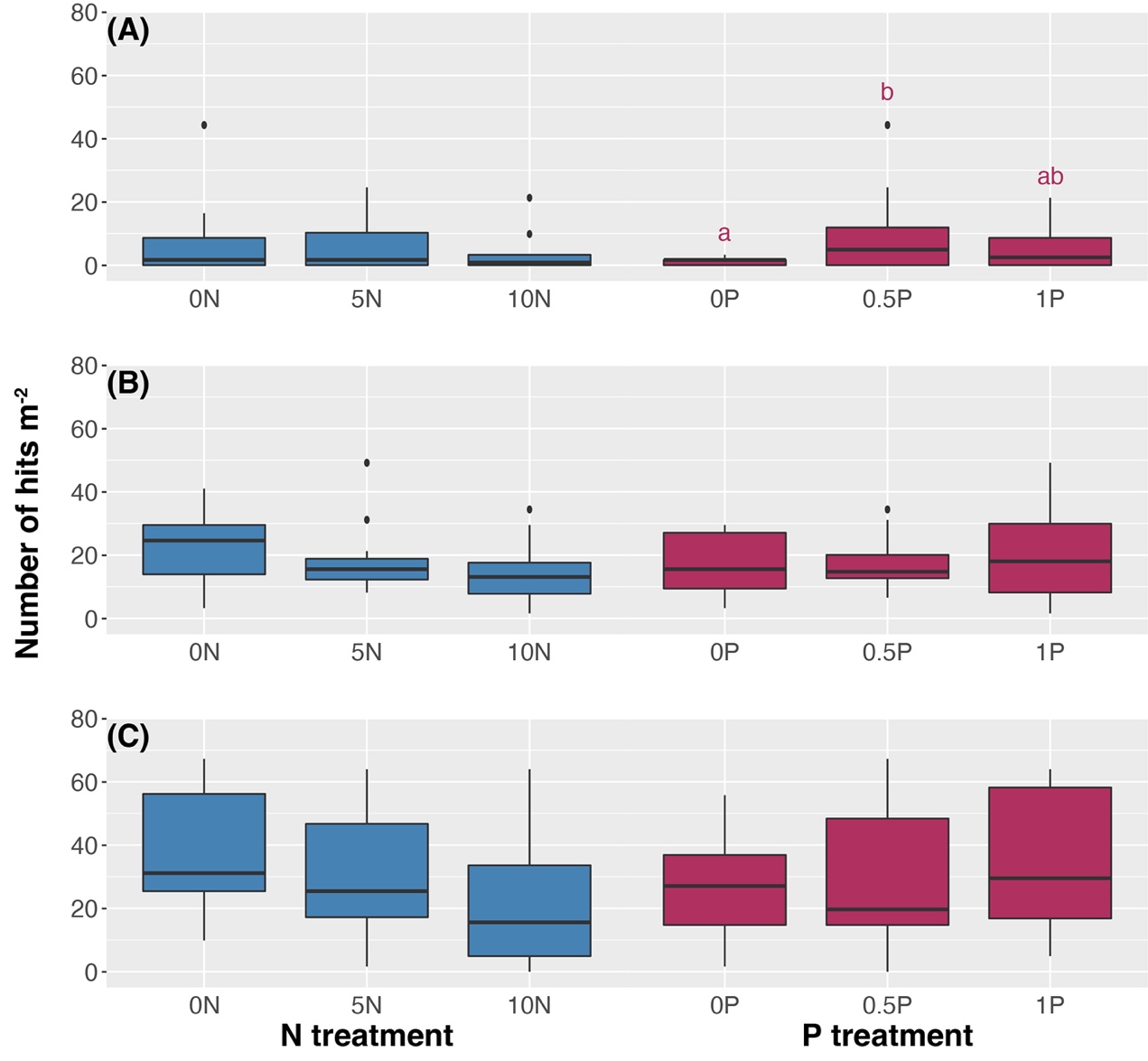
**

**Supplementary Figure S3** Abundance of *Chamaedaphne calyculata* (A), *Rhododendron tomentosum* (B) and *Vaccinium uliginosum* (C) in response to long-term N and/or P additions. Different uppercase letters represent significant differences (*P* < 0.05) among N treatments. Different lowercase letters represent significant differences (*P* < 0.05) among P treatments. Treatment abbreviations as described in Materials and Methods.

**
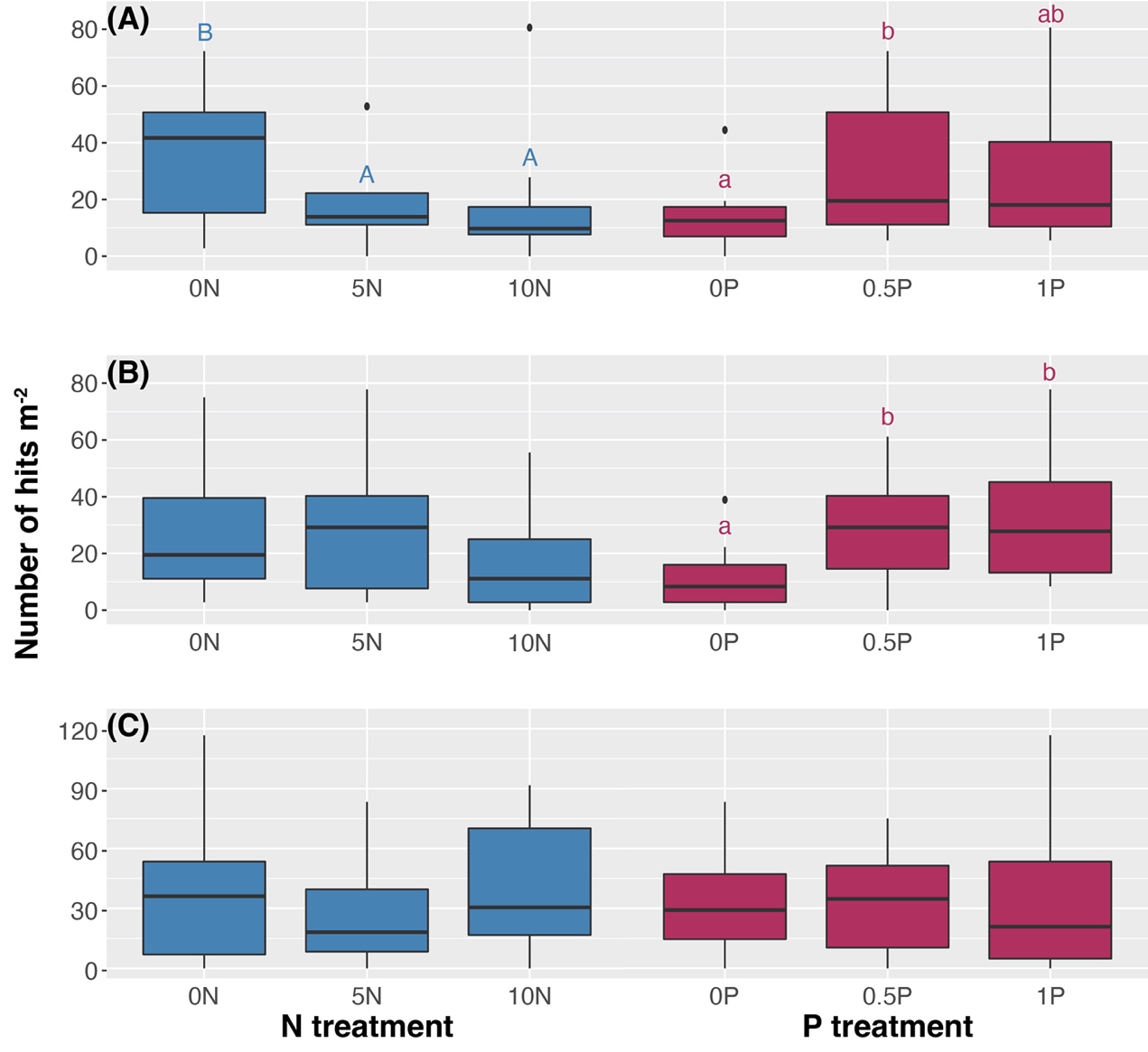
**

**Supplementary Figure S4** Relative abundance (mean ± SE) of *Clavaria sphagnicola* (A), *Galerina sphagnicola* (B), *Galerina paludosa* (C) and *Galerina tibiicystis* (D) in response to short-term N and/or P additions. Treatment abbreviations as described in Materials and Methods.

**
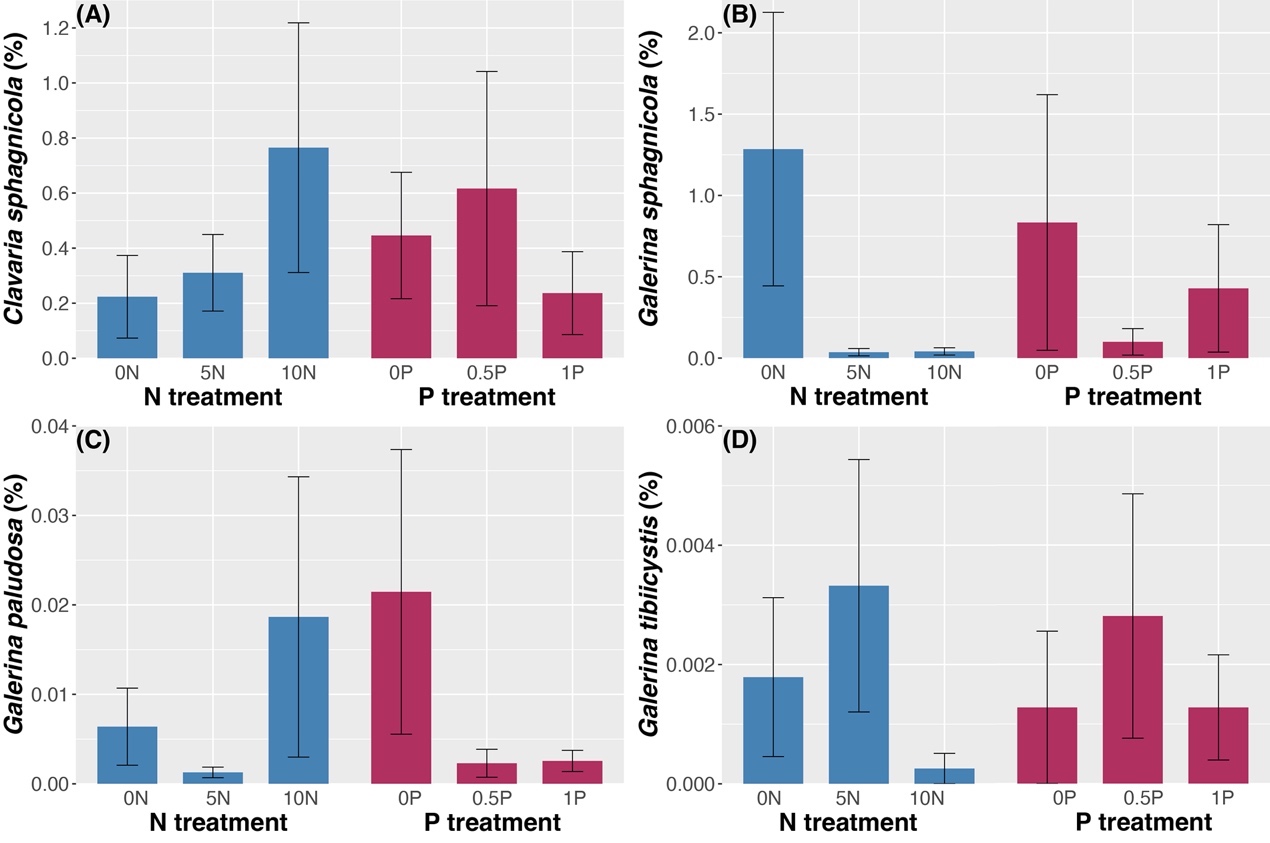
**

**Supplementary Figure S5** Relative abundance (mean ± SE) of *Clavaria sphagnicola* (A), *Galerina sphagnicola* (B), *Galerina paludosa* (C) and *Galerina tibiicystis* (D) in response to long-term N and/or P additions. Different uppercase letters represent significant differences (*P* < 0.05) among N treatments. Different lowercase letters represent significant differences (*P* < 0.05) among P treatments. Treatment abbreviations as described in Materials and Methods.

**
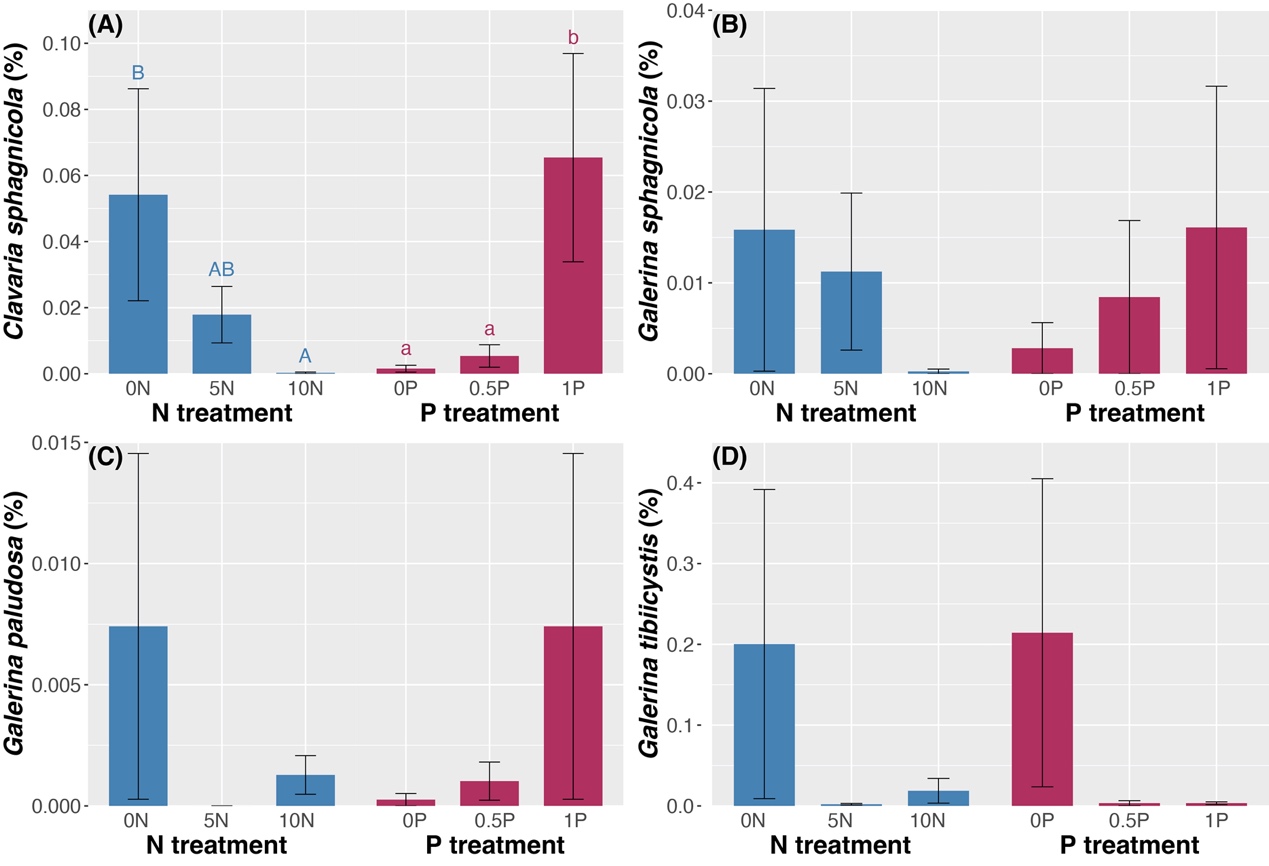
**

**Supplementary Table S1.** Fertilization experiment set-up.

| Fertilization treatment | N level (g m^-2^ yr^-1^) | P level (g m^-2^ yr^-1^) |
| --- | --- | --- |
| 1 | 0 | 0 |
| 2 | 5 | 0 |
| 3 | 10 | 0 |
| 4 | 0 | 0.5 |
| 5 | 5 | 0.5 |
| 6 | 10 | 0.5 |
| 7 | 0 | 1 |
| 8 | 5 | 1 |
| 9 | 10 | 1 |

The short- and long-term fertilization experiments have the same set-up.

**Supplementary Table S2.** Indicator species analysis for fungal operational taxonomic units (OTUs) among different N and/or P treatments. Indictor values are ordered from highest to lowest within each addition level.

| **OTU #** | **Indicator specificity** | **Indicator fidelity** | **Indicator value** | ***P*-value** | **Treatment** | **Taxonomy^a^** | **Functional group^b^** |
| --- | --- | --- | --- | --- | --- | --- | --- |
| ***Short-term experiment*** | | | | | | | |
| OTU45 | 0.8335 | 0.9167 | 0.874 | 0.0322 | 0N | *Coniochaeta* spp. | Saprotroph-Foliar endophyte-Plant pathogen-Animal pathogen-Lichen Parasite |
| OTU747 | 0.7565 | 1 | 0.87 | 0.0202 | 0N | *Helotiales* | Uncertain |
| OTU104 | 0.9288 | 0.75 | 0.835 | 0.0231 | 0N | *Chytridium olla* | Plant pathogen |
| OTU294 | 0.6957 | 0.6667 | 0.681 | 0.0213 | 0N | *Rhizophydiales* | Uncertain |
| OTU321 | 0.6579 | 0.6667 | 0.662 | 0.0235 | 0N | *Idriella* spp. | Saprotroph-Root endophyte |
| OTU291 | 0.7353 | 0.5833 | 0.655 | 0.0128 | 0N | *Curvularia tsudae* | Plant pathogen |
| OTU214 | 0.9833 | 0.3333 | 0.573 | 0.0221 | 0N | *Chytridium olla* | Plant pathogen |
| OTU39 | 0.8811 | 0.9167 | 0.899 | 0.0435 | 5N | *Ostropales* | Uncertain |
| OTU132 | 0.944 | 0.75 | 0.841 | 0.0085 | 5N | *Diversispora* spp. | Arbuscular Mycorrhizal |
| OTU19 | 0.6575 | 1 | 0.811 | 0.0305 | 5N | *Tomentella ellisii* | Ectomycorrhizal |
| OTU319 | 0.8481 | 0.75 | 0.798 | 0.0017 | 5N | Ascomycota | Uncertain |
| OTU121 | 0.6583 | 0.9167 | 0.777 | 0.0445 | 5N | *Epicoccum proteae* | Plant pathogen-Saprotroph-Foliar endophyte-Mycoparasite-Lichen Parasite |
| OTU307 | 0.875 | 0.6667 | 0.764 | 0.0013 | 5N | *Hypocreales* | Uncertain |
| OTU175 | 0.6987 | 0.8333 | 0.763 | 0.0154 | 5N | Ascomycota | Uncertain |
| OTU165 | 0.9531 | 0.5833 | 0.746 | 0.0194 | 5N | *Fusarium ramigenum* | Plant pathogen-Saprotroph-Foliar endophyte-Animal pathogen-Lichen Parasite |
| OTU117 | 0.597 | 0.9167 | 0.74 | 0.042 | 5N | *Idriella* spp. | Saprotroph-Root endophyte |
| OTU899 | 0.7937 | 0.6667 | 0.727 | 0.0284 | 5N | *Setomelanomma holmii* | Plant pathogen-Saprotroph |
| OTU360 | 0.7778 | 0.6667 | 0.72 | 0.0099 | 5N | Chytridiomycota | Uncertain |
| OTU273 | 0.6716 | 0.75 | 0.71 | 0.0234 | 5N | *Idriella* spp. | Saprotroph-Root endophyte |
| OTU364 | 0.7907 | 0.5833 | 0.679 | 0.0244 | 5N | Ascomycota | Uncertain |
| OTU622 | 0.8571 | 0.5 | 0.655 | 0.0094 | 5N | *Tremellales* | Uncertain |
| OTU478 | 1 | 0.3333 | 0.577 | 0.024 | 5N | *Funneliformis geosporum* | Arbuscular mycorrhizal |
| OTU201 | 0.7075 | 1 | 0.841 | 0.0002 | 10N | *Aureobasidium pullulans* | Plant pathogen-Saprotroph-Foliar endophyte-Epiphyte-Animal pathogen |
| OTU143 | 0.6205 | 0.9167 | 0.754 | 0.0048 | 10N | *Tremellaceae* | Uncertain |
| OTU465 | 0.6923 | 0.5 | 0.588 | 0.0488 | 10N | *Diaporthe betulae* | Plant pathogen-Saprotroph-Foliar endophyte |
| OTU702 | 0.7143 | 0.4167 | 0.546 | 0.0298 | 0P | *Funneliformis geosporum* | Arbuscular mycorrhizal |
| OTU243 | 0.907 | 0.9167 | 0.912 | 0.0476 | 0.5P | *Tomentella lapida* | Ectomycorrhizal |
| OTU268 | 0.7778 | 0.8333 | 0.805 | 0.0021 | 0.5P | *Hongkongmyces thailandicus* | Animal pathogen |
| OTU87 | 0.6393 | 1 | 0.8 | 0.0151 | 0.5P | *Paraphaeosphaeria xanthorρeae* | Saprotroph |
| OTU257 | 0.8291 | 0.5833 | 0.695 | 0.0153 | 0.5P | *Agaricales* | Uncertain |
| OTU308 | 0.8448 | 0.5 | 0.65 | 0.0328 | 0.5P | *Funneliformis geosporum* | Arbuscular mycorrhizal |
| OTU321 | 0.6053 | 0.6667 | 0.635 | 0.0314 | 0.5P | *Idriella* spp. | Saprotroph-Root endophyte |
| OTU528 | 0.6923 | 0.5 | 0.588 | 0.0323 | 0.5P | *Dothideomycetes* | Uncertain |
| OTU1336 | 1 | 0.3333 | 0.577 | 0.0257 | 0.5P | *Curvularia tsudae* | Plant pathogen |
| OTU787 | 1 | 0.3333 | 0.577 | 0.026 | 0.5P | *Diversispora spurca* | Arbuscular mycorrhizal |
| OTU13 | 0.6785 | 1 | 0.824 | 0.0179 | 1P | *Hyaloscyphaceae* | Saprotroph-Ericoid mycorrhizal-Ectomycorrhizal |
| OTU375 | 0.5588 | 0.8333 | 0.682 | 0.0144 | 1P | *Arnium* spp. | Saprotroph |
| ***Long-term experiment*** | | | | | | | |
| OTU34 | 0.9566 | 1 | 0.978 | 0.0004 | 0N | *Pseudoplectania nigrella* | Saprotroph |
| OTU50 | 0.8665 | 1 | 0.931 | 0.0167 | 0N | *Cladosporium flabelliforme* | Plant pathogen |
| OTU180 | 0.9007 | 0.75 | 0.822 | 0.049 | 0N | *Lecanicillium* spp. | Animal parasite |
| OTU69 | 0.6258 | 1 | 0.791 | 0.0237 | 0N | *Cantharellales* | Uncertain |
| OTU186 | 0.9167 | 0.6667 | 0.782 | 0.0023 | 0N | *Byssocorticium* spp. | Ectomycorrhizal |
| OTU137 | 0.989 | 0.5833 | 0.76 | 0.0177 | 0N | *Phenoliferia psychrophila* | Saprotroph |
| OTU436 | 0.7886 | 0.6667 | 0.725 | 0.0234 | 0N | *Kazachstania pintolopesii* | Saprotroph |
| OTU554 | 0.8276 | 0.5833 | 0.695 | 0.0059 | 0N | *Gnomonia* spp. | Foliar endophyte-Saprotroph-Plant pathogen |
| OTU505 | 0.6364 | 0.75 | 0.691 | 0.048 | 0N | *Polyporales* | Uncertain |
| OTU568 | 0.7778 | 0.5833 | 0.674 | 0.0178 | 0N | *Idriella* spp. | Saprotroph-Root endophyte |
| OTU1454 | 0.8889 | 0.5 | 0.667 | 0.0061 | 0N | *Exobasidium inconspicuum* | Plant pathogen |
| OTU606 | 0.8837 | 0.5 | 0.665 | 0.0179 | 0N | *Candida albicans* | Animal pathogen |
| OTU791 | 1 | 0.4167 | 0.645 | 0.0055 | 0N | Ascomycota | Uncertain |
| OTU670 | 0.7059 | 0.5833 | 0.642 | 0.0225 | 0N | *Dothideomycetes* | Uncertain |
| OTU285 | 0.8121 | 0.5 | 0.637 | 0.0353 | 0N | *Pezizales* | Uncertain |
| OTU409 | 0.9725 | 0.4167 | 0.637 | 0.037 | 0N | *Lophium arboricola* | Saprotroph |
| OTU365 | 0.9714 | 0.4167 | 0.636 | 0.0311 | 0N | *Pleosporales* | Uncertain |
| OTU778 | 0.7812 | 0.5 | 0.625 | 0.0152 | 0N | *Herpotrichiellaceae* | Saprotroph-Animal pathogen-Mycoparasite |
| OTU698 | 0.8519 | 0.4167 | 0.596 | 0.045 | 0N | *Tremellales* | Uncertain |
| OTU914 | 1 | 0.3333 | 0.577 | 0.0251 | 0N | *Hypocreales* | Uncertain |
| OTU272 | 0.7578 | 0.75 | 0.754 | 0.0462 | 5N | *Cantharellales* | Uncertain |
| OTU594 | 0.8182 | 0.5 | 0.64 | 0.0096 | 5N | *Tausonia pullulans* | Saprotroph |
| OTU653 | 1 | 0.3333 | 0.577 | 0.0257 | 5N | *Kockovaella litseae* | Epiphyte-Saprotroph-Foliar endophyte |
| OTU1469 | 0.9375 | 0.3333 | 0.559 | 0.0453 | 5N | *Exophiala attenuata* | Animal parasite-Saprotroph-Dark septate root endophyte |
| OTU58 | 0.9256 | 0.9167 | 0.921 | 0.0009 | 10N | *Saitozyma podzolica* | Saprotroph |
| OTU93 | 0.7842 | 1 | 0.886 | 0.0258 | 10N | *Sordariales* | Uncertain |
| OTU276 | 0.783 | 0.75 | 0.766 | 0.006 | 10N | *Sordariales* | Uncertain |
| OTU317 | 0.9172 | 0.5833 | 0.731 | 0.0167 | 10N | Ascomycota | Uncertain |
| OTU325 | 0.6727 | 0.75 | 0.71 | 0.0071 | 10N | *Podospora prolifica* | Saprotroph-Foliar endophyte |
| OTU763 | 0.7358 | 0.6667 | 0.7 | 0.0074 | 10N | Ascomycota | Uncertain |
| OTU295 | 0.9765 | 0.5 | 0.699 | 0.0039 | 10N | *Chytridiomycetes* | Uncertain |
| OTU516 | 0.7143 | 0.6667 | 0.69 | 0.0111 | 10N | *Preussia persica* | Saprotroph |
| OTU182 | 0.9414 | 0.5 | 0.686 | 0.0375 | 10N | *Dothideomycetes* | Uncertain |
| OTU1396 | 1 | 0.4167 | 0.645 | 0.0063 | 10N | *Genolevuria armeniaca* | Saprotroph-Epiphyte |
| OTU788 | 0.7083 | 0.5833 | 0.643 | 0.0344 | 10N | *Cyphellophora sessilis* | Saprotroph-Animal pathogen |
| OTU2013 | 1 | 0.3333 | 0.577 | 0.0256 | 10N | *Tremellales* | Uncertain |
| OTU696 | 1 | 0.3333 | 0.577 | 0.023 | 10N | *Xylariales* | Uncertain |
| OTU898 | 1 | 0.3333 | 0.577 | 0.0261 | 10N | *Sordariomycetes* | Uncertain |
| OTU514 | 0.9706 | 0.3333 | 0.569 | 0.0253 | 10N | *Pleotrichocladium opacum* | Saprotroph |
| OTU481 | 0.7525 | 0.4167 | 0.56 | 0.0411 | 10N | *Sistotrema autumnale* | Ectomycorrhizal |
| OTU49 | 0.95 | 1 | 0.975 | 0.0002 | 0P | *Hyaloscyphaceae* | Saprotroph-Ericoid mycorrhizal-Ectomycorrhizal |
| OTU19 | 0.9844 | 0.9167 | 0.95 | 0.0112 | 0P | *Diversispora spurca* | Arbuscular mycorrhizal |
| OTU30 | 0.8997 | 1 | 0.949 | 0.0459 | 0P | *Helotiales* | Uncertain |
| OTU56 | 0.8875 | 1 | 0.942 | 0.0012 | 0P | *Talaromyces stollii* | Saprotroph |
| OTU37 | 0.8736 | 1 | 0.935 | 0.0028 | 0P | *Sordariales* | Uncertain |
| OTU42 | 0.8247 | 1 | 0.908 | 0.0009 | 0P | *Phialocephala* spp. | Dark septate root endophyte |
| OTU328 | 0.9143 | 0.8333 | 0.873 | 0.0002 | 0P | *Curvularia tsudae* | Plant pathogen |
| OTU377 | 0.8846 | 0.8333 | 0.859 | 0.0002 | 0P | *Curvularia tsudae* | Plant pathogen |
| OTU146 | 0.7912 | 0.9167 | 0.852 | 0.0015 | 0P | *Scolecobasidium* spp. | Animal parasite-Plant pathogen-Foliar endophyte-Saprotroph |
| OTU71 | 0.8649 | 0.8333 | 0.849 | 0.006 | 0P | *Serendipitaceae* | Ericoid mycorrhizal |
| OTU61 | 0.9155 | 0.75 | 0.829 | 0.0364 | 0P | *Ostropales* | Uncertain |
| OTU916 | 0.7355 | 0.9167 | 0.821 | 0.0033 | 0P | *Paraphaeosphaeria angularis* | Saprotroph |
| OTU69 | 0.6723 | 1 | 0.82 | 0.0077 | 0P | *Cantharellales* | Uncertain |
| OTU143 | 0.6659 | 1 | 0.816 | 0.0063 | 0P | *Bryochiton* spp. | Plant pathogen-Saprotroph |
| OTU121 | 0.661 | 1 | 0.813 | 0.0038 | 0P | *Sanchytriaceae* | Uncertain |
| OTU211 | 0.7901 | 0.8333 | 0.811 | 0.0029 | 0P | *Physalospora vaccinii* | Plant pathogen |
| OTU60 | 0.9843 | 0.6667 | 0.81 | 0.016 | 0P | *Serendipita spp.* | Ericoid mycorrhizal |
| OTU773 | 0.9625 | 0.6667 | 0.801 | 0.0004 | 0P | *Archaeosporales* | Arbuscular mycorrhizal |
| OTU309 | 0.8969 | 0.6667 | 0.773 | 0.0082 | 0P | *Funneliformis geosporum* | Arbuscular mycorrhizal |
| OTU765 | 0.6346 | 0.9167 | 0.763 | 0.0017 | 0P | Ascomycota | Uncertain |
| OTU528 | 0.9679 | 1 | 0.984 | 0.0204 | 0.5P | *Cenococcum* spp. | Ectomycorrhizal |
| OTU39 | 0.8902 | 1 | 0.944 | 0.0444 | 0.5P | *Cladophialophora minutissima* | Saprotroph |
| OTU40 | 0.9976 | 0.75 | 0.865 | 0.0024 | 0.5P | *Herpotrichiellaceae* | Saprotroph-Animal pathogen-Mycoparasite |
| OTU116 | 0.8538 | 0.75 | 0.8 | 0.0351 | 0.5P | *Inocybe* spp. | Ectomycorrhizal |
| OTU238 | 0.8953 | 0.6667 | 0.773 | 0.0172 | 0.5P | *Idriella* spp. | Saprotroph-Root endophyte |
| OTU344 | 0.8182 | 0.5 | 0.64 | 0.0472 | 0.5P | Ascomycota | Uncertain |
| OTU927 | 0.8462 | 0.4167 | 0.594 | 0.0306 | 0.5P | *Clonostachys rosea* | Saprotroph-Plant pathogen-Mycoparasite-Animal parasite-Endophyte |
| OTU836 | 0.8 | 0.4167 | 0.577 | 0.0263 | 0.5P | *Pleosporales* | Uncertain |
| OTU31 | 0.9848 | 0.8333 | 0.906 | 0.0155 | 1P | *Hyaloscyphaceae* | Saprotroph-Ericoid mycorrhizal-Ectomycorrhizal |
| OTU77 | 0.7798 | 1 | 0.883 | 0.0025 | 1P | *Hyaloscyphaceae* | Saprotroph-Ericoid mycorrhizal-Ectomycorrhizal |
| OTU506 | 0.7239 | 1 | 0.851 | 0.0367 | 1P | Ascomycota | Uncertain |
| OTU246 | 0.7076 | 1 | 0.841 | 0.0053 | 1P | *Lecanicillium fusisporum* | Animal pathogen-Foliar endophyte |
| OTU54 | 0.993 | 0.6667 | 0.814 | 0.0243 | 1P | *Sordariomycetes* | Uncertain |
| OTU843 | 0.8636 | 0.6667 | 0.759 | 0.0009 | 1P | *Ramichloridium apiculatum* | Plant pathogen-Foliar endophyte |
| OTU484 | 0.7273 | 0.75 | 0.739 | 0.0017 | 1P | Cladophialophora spp. | Saprotroph-Dark septate root endophyte-Mycoparasite |
| OTU1328 | 0.9231 | 0.5 | 0.679 | 0.0104 | 1P | *Leotiomycetes* | Uncertain |
| OTU216 | 0.9144 | 0.5 | 0.676 | 0.029 | 1P | *Polyscytalum neofecundissimum* | Saprotroph-Animal pathogen |
| OTU316 | 0.9046 | 0.5 | 0.673 | 0.0261 | 1P | *Clavaria sphagnicola* | Saprotroph-Biotroph-CHEGD symbiotroph |
| OTU279 | 1 | 0.4167 | 0.645 | 0.0078 | 1P | Rozellomycota | Uncertain |
| OTU176 | 0.996 | 0.4167 | 0.644 | 0.0161 | 1P | *Acarosporales* | Uncertain |
| OTU196 | 0.9609 | 0.4167 | 0.633 | 0.0396 | 1P | *Epibryon interlamellare* | Plant pathogen |
| OTU277 | 0.9518 | 0.4167 | 0.63 | 0.022 | 1P | *Monocillium constrictum* | Saprotroph |
| OTU601 | 1 | 0.3333 | 0.577 | 0.0219 | 1P | *Spiculogloeales* | Uncertain |
| OTU794 | 1 | 0.3333 | 0.577 | 0.0236 | 1P | *Nectriopsis fuliginicola* | Lichen parasite-Protistan parasite |
| OTU930 | 0.7692 | 0.4167 | 0.566 | 0.0256 | 1P | *Microdochiaceae* | Saprotroph-Root endophyte |

**Supplementary Table S3.** Outputs of *rdacca.hp*.

| **Variables** | **Unique** | **Shared**  **effect**^1^ | **Individual**  **importance** | **Individual**  **percent**^2^ |
| --- | --- | --- | --- | --- |
| *Short-term experiment* | | | | |
| TN | 0.0106 | 0.0023 | 0.0129 | 13.18 |
| TC | 0.0089 | 0.0001 | 0.0090 | 9.19 |
| Water content | 0.0007 | 0.0105 | 0.0112 | 11.44 |
| 1630/1090 | -0.0057 | 0.0112 | 0.0055 | 5.62 |
| Evergreen shrubs | 0.0278 | 0.0023 | 0.0301 | 30.75 |
| Deciduous shrubs | 0.0099 | 0.0017 | 0.0116 | 11.85 |
| Graminoid | 0.0149 | -0.0094 | 0.0055 | 5.62 |
| *Sphagnum* | 0.0190 | -0.0069 | 0.0121 | 12.36 |
| Total | 0.0861 | 0.0118 | 0.0979 | 100.01 |
|  |  |  |  |  |
| *Long-term experiment* | | | | |
| TN | 0.0031 | 0.0053 | 0.0084 | 7.78 |
| TP | 0.0054 | 0 | 0.0054 | 5 |
| TC | 0.0249 | -0.0102 | 0.0147 | 13.62 |
| K | 0.0134 | -0.0065 | 0.0069 | 6.39 |
| 1420/1090 | 0.0075 | 0.0062 | 0.0137 | 12.7 |
| 1510/1090 | -0.0017 | 0.0066 | 0.0049 | 4.54 |
| 1630/1090 | 0.0031 | 0.0092 | 0.0123 | 11.4 |
| 1720/1090 | 0.0013 | 0.0015 | 0.0028 | 2.59 |
| Evergreen shrubs | 0.0102 | -0.0014 | 0.0088 | 8.16 |
| Deciduous shrubs | 0.0155 | 0.0052 | 0.0207 | 19.18 |
| Graminoid | 0.0023 | -0.0004 | 0.0019 | 1.76 |
| *Sphagnum* | 0.0045 | 0.0029 | 0.0074 | 6.86 |
| Total | 0.0895 | 0.0184 | 0.1079 | 100 |

^1^Total average of shared effect with other predictors.

^2^Individual effect divided by total adjusted *R*^2^ from ‘Individual importance’.

Variables with negative individual importance have been removed.

**Supplementary Table S4.** Spearman’s correlation between relative abundance of different fungal functional guilds and environmental factors. Spearman’s rank correlation coefficients are shown with *P* values in paratheses. Significant (*P* < 0.05) coefficients are highlighted in bold.

|  | TN | TP | TC | K | | SWC | 1420/1090 | 1510/1090 | 1630/1090 | 1720/1090 |
| --- | --- | --- | --- | --- | --- | --- | --- | --- | --- | --- |
| *Short-term experiment* | | | | |  |  |  |  |  |  |
| ErMF | 0.17  (0.315) | 0.16  (0.349) | 0.25  (0.145) | -0.13  (0.436) | | -0.05  (0.782) | 0.01  (0.956) | -0.03  (0.879) | 0.05  (0.787) | -0.17  (0.316) |
| EcMF | 0.22  (0.192) | 0.16  (0.354) | -0.02  (0.917) | 0.31  (0.065) | | -0.20  (0.233) | 0.19  (0.275) | 0.32  (0.054) | **0.34**  (0.044) | **0.50**  (0.001) |
| AMF | -0.21  (0.225) | 0.003  (0.988) | -0.23  (0.185) | -0.06  (0.718) | | **0.35**  (0.038) | -0.04  (0.802) | -0.16  (0.364) | **-0.46**  (0.005) | -0.21  (0.203) |
| Ligno | -0.26  (0.124) | **0.50**  (0.002) | 0.15  (0.386) | -0.06  (0.713) | | -0.03  (0.848) | 0.18  (0.297) | 0.23  (0.176) | -0.01  (0.955) | 0.20  (0.245) |
| Sapro | -0.27  (0.111) | -0.11  (0.512) | 0.29  (0.084) | 0.22  (0.203) | | -0.10  (0.570) | -0.15  (0.374) | -0.05  (0.780) | -0.03  (0.848) | 0.01  (0.945) |
| Sphag | 0.16  (0.337) | **0.43**  (0.009) | 0.03  (0.895) | 0.04  (0.814) | | **-0.42**  (0.011) | 0.13  (0.443) | 0.23  (0.174) | 0.25  (0.139) | 0.26  (0.131) |
| *Long-term experiment* | | | | |  |  |  |  |  |  |
| ErMF | -0.22  (0.207) | -0.05  (0.771) | 0.23  (0.185) | 0.13  (0.446) | | 0.16  (0.336) | 0.03  (0.861) | 0.12  (0.488) | 0.11  (0.536) | -0.06  (0.720) |
| EcMF | 0.06  (0.715) | 0.15  (0.375) | **-0.43**  (0.009) | -0.16  (0.355) | | -0.03  (0.869) | -0.07  (0.666) | 0.16  (0.346) | **0.34**  (0.043) | 0.14  (0.414) |
| AMF | -0.13  (0.440) | -0.12  (0.492) | -0.24  (0.158) | 0.04  (0.796) | | 0.03  (0.851) | -0.03  (0.841) | -0.01  (0.968) | -0.12  (0.499) | -0.07  (0.703) |
| Ligno | -0.04  (0.817) | 0.06  (0.734) | -0.10  (0.560) | 0.31  (0.062) | | -0.03  (0.852) | -0.22  (0.206) | -0.08  (0.639) | -0.13  (0.458) | -0.18  (0.281) |
| Sapro | -0.02  (0.912) | -0.06  (0.734) | -0.05  (0.759) | 0.19  (0.263) | | **-0.46**  (0.005) | 0.07  (0.694) | 0.02  (0.892) | -0.02  (0.887) | -0.06  (0.713) |
| Sphag | -0.22  (0.200) | 0.05  (0.780) | -0.08  (0.648) | 0.30  (0.080) | | 0.30  (0.074) | -0.11  (0.509) | -0.08  (0.634) | -0.09  (0.612) | -0.08  (0.643) |

ErMF, ericoid mycorrhizal fungi; EcMF, ectomycorrhizal fungi; AMF, arbuscular mycorrhizal fungi; Ligno, lignocellulose degrading fungi; Sapro, saprotrophic fungi; Sphag, *Sphagnum*-associated fungi; TN, total nitrogen concentration; TP, total phosphorus concentration; TC, total carbon concentration; K, potassium concentration; 1420/1090, 1510/1090, 1630/1090 and 1720/1090 are humification indices. See Supplementary Methods for details.
